# Quadrature-free immersed isogeometric analysis

**DOI:** 10.1007/s00366-022-01644-3

**Published:** 2022-04-25

**Authors:** P. Antolin, T. Hirschler

**Affiliations:** Institute of Mathematics, Chair of Numerical Modelling and Simulation, École Polytechnique Fédérale de Lausanne, Lausanne, Switzerland

**Keywords:** Immersed methods, Computer-Aided Design, Isogeometric analysis, Quadrature-free

## Abstract

This paper presents a novel method for solving partial differential equations on three-dimensional CAD geometries by means of immersed isogeometric discretizations that do not require quadrature schemes. It relies on a newly developed technique for the evaluation of polynomial integrals over spline boundary representations that is exclusively based on analytical computations. First, through a consistent polynomial approximation step, the finite element operators of the Galerkin method are transformed into integrals involving only polynomial integrands. Then, by successive applications of the divergence theorem, those integrals over B-Reps are transformed into the first surface and then line integrals with polynomials integrands. Eventually, these line integrals are evaluated analytically with machine precision accuracy. The performance of the proposed method is demonstrated by means of numerical experiments in the context of 2D and 3D elliptic problems, retrieving optimal error convergence order in all cases. Finally, the methodology is illustrated for 3D CAD models with an industrial level of complexity.

## Introduction

The integration of Computer-Aided Design (CAD) and Computer-Aided Engineering has gained interest during the last two decades with the introduction of new numerical approaches as, for instance, the isogeometric paradigm [1, 2] or meshfree strategies [3]. Particularly, spline-based geometric models have been found to present excellent performance for numerical simulations [4–7]. This opens the door to the formation of all-in-one design frameworks where a single geometric model is simultaneously used for parameterizing the shape of the object of interest and performing advanced numerical analyses [8–11]. The combination into one single model of both high-fidelity geometrical properties and efficient analysis performances is, however, far from trivial in general. Indeed, generating analysis-suitable geometric models for complex industrial designs requires advanced numerical tools. To achieve this goal, two different strategies can be undertaken: The first one consists in generating a fully boundary-conforming and matching geometric model such that standard analysis procedures can be directly applied. Generating those spline meshes is however a quite challenging task for geometries with complex topologies, especially when only tensor-product splines are considered [12–15]. For those cases, unstructured spline meshes [16–20] constitute an appealing alternative. On the contrary, the second approach aims to directly use standard CAD models which may contain non-conforming and trimmed surfaces and present geometric defects, such as water leaks or surface overlaps, and require the use of high-end analysis procedures [21–26]. Interested readers may refer to [27], and the many references therein, for an extensive review in the context of isogeometric methods. The present work falls into this second category.

A major ingredient that is commonly required to perform numerical analyses over CAD models is an efficient integration procedure which enables to evaluate integrals over complex domains such as curved polyhedrons. This is, for instance, the case when employing non-conformal analysis methods, where the geometric representation is decoupled from the discretization of the solution [28–34].

In this context of immersed and enriched FEM, there exist several integration approaches. In 3D, among the most common ones is worth highlighting *octree subdivision* [35–38] which consists in adaptively subdividing the domain of integration into sub-cells (voxels in 3D, or simple pixels in 2D). The obtained piecewise constant approximation of the underlying geometry can be improved by performing a local boundary reparameterization at the finest level of this recursion procedure via a (low-order) tessellation method [39, 40]. Despite the beneficial simplicity and robustness of this decomposition-based method, it may suffer from high computational cost due to the large number of integration sub-cells, especially in three-dimensional and high-order methods.

For problems where the geometric representation of the boundary is of major importance, alternative approaches are considered. They consist in generating boundary-conforming sub-meshes which are generally non-analysis-suitable (due to the presence of hanging nodes, missing connectivity, singularities, etc.) but which are handy for integration purposes. The high-fidelity representation of the geometry boundaries, even for complex geometries, yields a high-accuracy in the evaluation of integrals. Nonetheless, even if the difficulty of generating such a high-order mesh is lower than building fully analysis-suitable boundary-conforming parameterizations, it still remains a challenging and time consuming task for complex 3D geometries. On the other hand, for two-dimensional geometries, the problem can be usually solved in a more accurate way: We refer the interested reader to the extensive survey [27].

An appealing alternative to these two approaches is the use of *moment fitting* techniques [41–44] in which coarse, but accurate, quadrature rules are generated for complex integration domains by tuning the positions and/or weights of the quadrature points. Nevertheless, these methods come at a price: The creation of tailored quadrature rules requires the computation of polynomial integrals over complex domains at a pre-processing stage, which calls for the use of alternative integration techniques.

Finally, there exists a fourth group of strategies for computing integrals over curved polyhedrons that lies in deriving dedicated integration rules for specific classes of integrands, as for instance polynomial functions. Indeed, it is known that integrating polynomials and other homogeneous functions over (curved) polyhedrons can be done more efficiently by invoking the divergence theorem [45–49]. These results can be exploited in several ways: One can perform a *polynomial approximation* of the integrands of interest such that the integration can be done straightforwardly [50–52]; those specific rules can be applied at the pre-processing stage of *moment-fitting* methods [42, 43, 53]; or can be used for creating quadrature schemes on the edges or faces of the polyhedrons for integrating the involved operators [54, 55].

Within this category, worth mentioning are the recent works [49, 55], where the divergence theorem is used for transforming volumetric integrals into either surface or line integrals. In [55], the authors reduced 3D integrals of general functions to 1D integrals, that are finally evaluated using fine quadrature rules. This extends the previous work [56] for the case of 2D geometries. Similarly, in [49] the complexity of 3D integrals is reduced to just vertices evaluations in the case of planar polyhedra. For the case of B-reps composed of Bézier triangles or non-trimmed B-splines patches, the authors in [49] applied the divergence theorem just once, transforming 3D integrals into 2D ones, which are approximated through standard quadrature rules.

Aligned with these ideas, in this work we present a fully *quadrature-free* method for integrating polynomials over general B-rep models enclosed by trimmed spline surfaces. The procedure is based on two successive applications of the divergence theorem, reducing volumetric integrals to the first surface and then line integrals, that are computed analytically up to machine precision. Hence, this can be seen as a generalization of those previous works, eliminating the need of quadrature rules. Such an approach is particularly well suited to B-Rep models as it only uses a description of the boundaries. On the other hand, handling B-Rep models with *octree subdivision* methods may be cumbersome as they have to evaluate if a point in the Euclidean space lies inside or outside the body for every single quadrature point, what is not always trivial.

Furthermore, we show how this integration procedure, combined with a consistent polynomial approximation step, leads to a new analysis tool for immersed isogeometric methods that skips the need of complex quadrature rules. This new integration procedure is highly-accurate (up to surface-surface intersection errors), and thus enables to handle analysis over high-order discretizations. In comparison, it is known that low-order approaches, as for instance, octree methods, require many quadrature points to keep the consistency error below the discretization error such that optimal convergence rates are retained in simulations. Consequently, it leads to high computational costs in general, which drastically reduces the benefits of employing high-order discretizations.

**Fig. 1:**
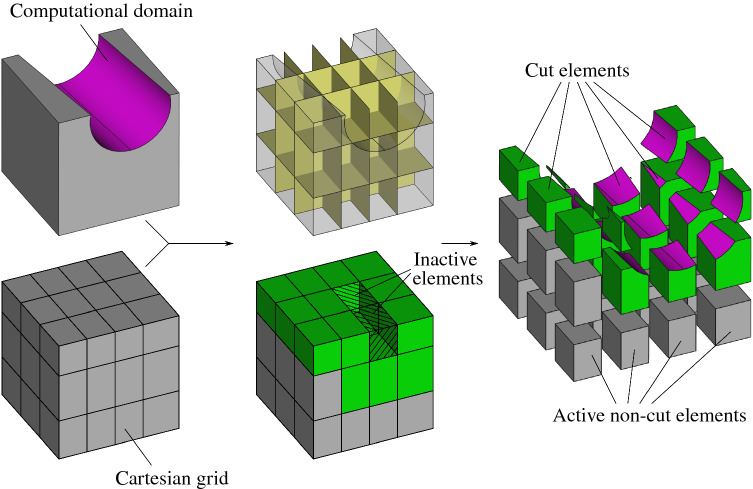
Immersed methods setting

The developed approach is presented as follows: We firstly introduce in Sect. 2 the basics regarding immersed isogeometric analysis to further detail the scope of application of this work, and describe a consistent approximation step required for transforming the involved integrands into polynomials. Then, in Sect. 3, we discuss the geometric modeling via splines, trimming, and boundary-representation, as commonly undertaken in CAD. In Sect. 4, the proposed *quadrature-free* integration over B-Reps is presented. Finally, in Sect. 5, we solve elliptic PDEs and perform several numerical experiments to confirm the accuracy of the approach. Lastly, concluding remarks are summarized in Sect. 6.

## Immersed isogeometric analysis

With the aim of introducing immersed methods, the used notation, and the main ideas behind this work, let us first introduce a classical Poisson’s problem as our driving example. Even if the problem is presented in a 3D context, the same ideas are directly applicable to 2D problems.

Let $\Omega \subset \mathbb {R}^3$ be the computational domain whose boundary is partitioned as $\Gamma _N\cup \Gamma _D=\partial \Omega$ and $\Gamma _N\cap \Gamma _D=\emptyset$. We also define a functional space $H^1_D(\Omega )=\{ v\in H^1(\Omega ): v\vert _{\Gamma _D} =0 \}$, such that the Poisson’s problem reads: find $u\in {H^1_D(\Omega )}$ solution of:

1 $$\begin{aligned} \begin{aligned} -\nabla \cdot ({\varvec{K}}\nabla {u}) = f&~~\text {in}~\Omega , \\ \nabla {u}\cdot {{\varvec{n}}}=g&~~\text {on}~\Gamma _N, \\ u=0&~~\text {on}~\Gamma _D, \end{aligned} \end{aligned}$$

where ${\varvec{K}}\in L^2(\Omega )^{3\times {3}}$ is the symmetric diffusivity operator; $f\in L^2(\Omega )$ and $g\in H^{-1/2}(\Gamma _N)$ are the source and Neumann terms, respectively; and ${{\varvec{n}}}\in \mathbb {R}^3(\partial \Omega )$ is the outward pointing unit normal on the boundary. For the sake of clarity, and without constituting any limitation, in the problem (1) and hereinafter we assume homogeneous Dirichlet boundary conditions on $\Gamma _D$.

The associated weak problem can be written as: find $u\in H^1_D(\Omega )$ such that

2 $$\begin{aligned} a(u,v) = {b}(v), \quad \forall {v}\in H^1_D(\Omega ), \end{aligned}$$

where

3 $$\begin{aligned} \begin{aligned} a(u,v)&= \int _{\Omega } \nabla {u}\cdot {\varvec{K}}\nabla {v} \,{\text {d}}\Omega ,\\ b(v)&= \int _{\Omega } f\,{v} \,{\text {d}}\Omega + \int _{\Gamma _N} g\,{v} \,{\text {d}}\Gamma . \end{aligned} \end{aligned}$$

### Immersed methods

The philosophy behind immersed methods is depicted in Fig. 1. It consists in embedding the computational domain $\Omega$ into a grid ${\mathcal {T}}_h(\Omega _0)$ of a larger domain $\Omega _0$, such that $\Omega \subset \Omega _0\subset \mathbb {R}^3$. The solution of the weak problem (3) is then discretized over a subset of the grid ${\mathcal {T}}_h(\Omega _0)$, which allows a decoupling of the solution discretization from the actual geometry. This simple and rather straightforward procedure is the one and only mesh generation task to undertake within immersed-like approaches, making this class of methods very appealing. Indeed, this can largely ease the design-to-analysis workflow since the computational domain can be directly prescribed as a geometric model with any representation commonly used in CAD, as for instance the Boundary-Representation (B-Rep), detailed in Sect. 3. In return, the price to pay during the analysis lies in the introduction of so-called cut or trimmed elements, as illustrated in Fig. 1. This requires the integration of quantities over cut elements (as discussed in the introduction, see Sect. 1). This work focuses on this particular challenge one would face when dealing with enriched or unfitted finite element methods over B-Rep models.

As the computational domain is $\Omega$ and not $\Omega _0$, the partition ${\mathcal {T}}_h(\Omega _0)$ is restricted to a subset ${\mathcal {T}}_h(\Omega )$ as:

4 $$\begin{aligned} {\mathcal {T}}_h(\Omega ): = \{ Q\ \vert \ Q\in {\mathcal {T}}_h({\Omega _0}) : Q\cap \Omega \ne \emptyset \}. \end{aligned}$$

Indeed, the grid ${\mathcal {T}}_h(\Omega _0)$ naturally splits the domain $\Omega _0$ into three complementary partitions of elements:

5a $$\begin{aligned} {\mathcal {T}\,}^\Gamma _h(\Omega ) :&= \{ Q\ \vert \ Q\in {\mathcal {T}}_h({\Omega }) : Q\cap \Omega \ne Q \}\,, \end{aligned}$$

5b $$\begin{aligned} {\mathcal {T}\,}^{\text {int}}_h(\Omega ) :&= \{ Q\ \vert \ Q\in {\mathcal {T}}_h({\Omega }) : Q\cap \Omega = Q \}\,, \end{aligned}$$

5c $$\begin{aligned} {\mathcal {T}\,}^0_h(\Omega _0) :&= \{ Q\ \vert \ Q\in {\mathcal {T}}_h({\Omega _0}) : Q\cap \Omega = \emptyset \}\,, \end{aligned}$$

such that ${\mathcal {T}}_h(\Omega )={\mathcal {T}\,}^{\text {int}}_h(\Omega )\cup {\mathcal {T}\,}^\Gamma _h(\Omega )$ and ${\mathcal {T}}_h(\Omega _0)={\mathcal {T}}_h(\Omega )\cup {\mathcal {T}\,}^0(\Omega _0)$. As depicted in Fig. 1, the elements belonging to these three subsets are denoted as cut, non-cut, and inactive elements, respectively.

In this work, we limit our discussion to the case of 3D immersed isogeometric methods, nevertheless, the presentation is kept rather general and can be easily adapted to generic immersed methods [28] or particular cases as, for instance, CutFEM [31] or Finite Cell Methods [57], among others.

To solve numerically the weak problem (3) we construct a discrete spline space $\mathbb {V}_h(\Omega _0)$ over the grid ${\mathcal {T}}_h(\Omega _0)$ as:

6 $$\begin{aligned} \mathbb {V}_h(\Omega _0) = \text {span}\{ N^p_i,~i\in \mathcal {I}_0\}, \end{aligned}$$

where $N^p_i$ denotes generic spline basis functions of degree $p>0$ and arbitrary continuity (up to $p-1$), and $\mathcal {I}_0$ is the set of indices of those basis functions. In this work we use tensor-product B-splines, but the extensions to other cases as, e.g., hierarchical splines [58] or T-splines [59], is straightforward. For the sake of simplicity, henceforward we drop the superscript *p* from $N^p_i$ and assume that the spline degree *p* is constant along the three parametric directions.

The support of some basis functions of the space $\mathbb {V}_h(\Omega _0)$ may not intersect the domain $\Omega$ and, consequently, they do not contribute to the solution of the problem (3). Therefore, we trim the space $\mathbb {V}_h(\Omega _0)$ as:

7 $$\begin{aligned} \mathbb {V}_h(\Omega ) = \text {span}\{ N_i\in \mathbb {V}_h(\Omega _0): \text {supp}\{ N_i \} \cap \Omega \ne \emptyset \}, \end{aligned}$$

that, as already studied in [9], holds optimal approximation properties. It is a well-known fact that the active support of some basis functions in $\mathbb {V}_h(\Omega )$ ($\text {supp}\{ N_i \} \cap \Omega$) may be small, which could yield ill-conditioned operators. This is an active research topic [27, 60–62] that exceeds the scope of this work.

Henceforward, we assume the Dirichlet boundary $\Gamma _D$ to be such that $\Gamma _D\subset \partial \Omega _0\cap \partial \Omega$, what grants the strong enforcement of Dirichlet boundary conditions. The opposite case ($\Gamma _D\not \subset \partial \Omega _0$) entails the imposition of Dirichlet conditions in a weak sense. We refer the interested reader to [63–65] for a dedicated discussion and to [62] for a study, in the case of spline spaces, of the inherent stability issues.

Thus, by means of the assumption $\Gamma _D\subset \partial \Omega _0\cap \partial \Omega$, we can define the space:

8 $$\begin{aligned} \mathbb {V}^D_h(\Omega ) = \{ v_h\in \mathbb {V}_h(\Omega ) : v_h\,\vert _{\Gamma _D} =0 \}. \end{aligned}$$

that allows us to discretize the continuous weak problem (3) as: find ${u}_h\in \mathbb {V}^D_h(\Omega )$ solution of:

9 $$\begin{aligned} a({u}_h,{v}_h) = {b}({v}_h), \quad \forall {v}_h\in {\mathbb {V}^D_h(\Omega )}, \end{aligned}$$

where the discrete versions of the bilinear form *a* and the linear form *b* are decomposed as:

10 $$ \begin{aligned} a(u_h,v_h) & =\sum _{Q\in {\mathcal {T}\,}^{\text {int}}_h(\Omega )} \int _{Q} \nabla {u_h}\cdot {\varvec{K}}\nabla {v_h} \,{\text {d}}Q \\ & + \sum _{Q\in {\mathcal {T}\,}^\Gamma _h(\Omega )} \int _{Q\cap \Omega } \nabla {u_h}\cdot {\varvec{K}}\nabla {v_h} \,{\text {d}}Q, \\ b(v_h) &= \sum_{Q \in {\mathcal {T}}^{\text{int}}_{h}(\Omega)} \int_{Q} f{v_h} \,{\text d}Q \\ & + \sum_{Q\in {\mathcal {T}}^{\Gamma}_h(\Omega)} \int_{Q\cap \Omega} f\,{v_h} \,{\text d}Q + \sum_{Q\in{\mathcal {T}}^{\text{int}}_h(\Omega)} \int_{Q\cap\Gamma_N} g\,{v_h} \,{\text d}\Gamma + \sum_{Q\in{\mathcal {T}}^{\Gamma}_h(\Omega)} \int_{Q\cap\Gamma_N} g\,{v_h} \,{\text d}\Gamma . \end{aligned}$$

The computation of the integrals over non-cut elements $Q\in {\mathcal {T}\,}^{\text {int}}_h(\Omega )$ is straightforward and can be performed using classical quadrature schemes. However, the evaluation of integrals over cut elements $Q\in {\mathcal {T}\,}^{\Gamma }_h(\Omega )$ is a challenging problem and one of the Achilles’ heels of isogeometric immersed methods in 3D (see the related discussion in Sect. 1). The main contribution of this article regards the computation of those integrals through a quadrature-free approach for the case of cut elements defined as B-Rep models. This procedure is presented in Sect. 4. Nonetheless, this method is only applicable to the case in which the integrands are polynomial functions. Thus, before introducing it, in the next section the integrals in (10) are transformed such as they only rely on polynomial integrands.

### Polynomial approximation of finite element operators

When considering spline discretizations over the grid ${\mathcal {T}}_h(\Omega )$, the terms $\nabla {u_h}$, $\nabla {v_h}$, and $v_h$ in the operators (10) take polynomial forms $\forall Q\in {\mathcal {T}}_h(\Omega )$. On the contrary, the datum quantities involved (i.e., ${\varvec{K}}$, *f*, and *g*) may not be polynomials in general.

Hence, to work with integrals that only present polynomial integrands, we seek to exploit a key result introduced in [66]: It is possible to perform a polynomial approximation of the integrands in (10) without deteriorating the solution. More specifically, instead of solving the problem (9), we consider the following approximate problem: find $\bar{u}_h\in \mathbb {V}^D_h(\Omega )$ solution of:

11 $$\begin{aligned} \bar{a}(\bar{u}_h,{v}_h) = \bar{b}({v}_h), \quad \forall {v}_h\in \mathbb {V}^D_h(\Omega ), \end{aligned}$$

where the discrete forms in (10) are replaced by:

12 $$\begin{aligned} \bar{a}(\bar{u}_h,v_h)&= \sum _{Q\in {\mathcal {T}\,}^{\text {int}}_h(\Omega )} \int _{Q} \nabla \bar{u}_h\cdot \bar{{\varvec{K}}}\nabla {v_h} \,{\text {d}}Q \\&\quad + \sum _{Q\in {\mathcal {T}\,}^\Gamma _h(\Omega )} \int _{Q\cap \Omega } \nabla \bar{u}_h\cdot \bar{{\varvec{K}}}\nabla {v_h} \,{\text {d}}Q,\\ \bar{b}(v_h) =& \sum_{Q\in {\text{T}}^{\text{int}}_h(\Omega)} \int_{Q} \bar{f}\,{v_h} \,{\text{d}}Q + \sum_{Q\in{\text{T}}^{\Gamma}_h(\Omega)} \int_{Q\cap\Omega} \bar{f}\,{v_h} \,{\text{d}}Q + \sum_{Q\in{\text{T}}^{\text{int}}_h(\Omega)} \int_{Q\cap\Gamma_N} \bar{g}\,{v_h} \,{\text{d}} \Gamma + \sum_{Q\in{\text{T}}^{\Gamma}_h(\Omega)} \int_{Q\cap\Gamma_N} \bar{g}\,{v_h} \,{\text{d}}\Gamma, \end{aligned}$$

that involves the following polynomial approximations:

13 $$\begin{aligned} \bar{{\varvec{K}}} = \Pi ^{h}{{\varvec{K}}},\qquad \bar{f} = \Pi ^{h}{f},\qquad \bar{g} = \Pi ^{h}{g}. \end{aligned}$$

In the approximations above, the projection spaces must be chosen carefully, such that the introduced consistency errors do not pollute the numerical solution. Thus, by recalling [66, Theorem 13], we know that the projection of ${\varvec{K}}$, *f*, and *g* into spline spaces of degree $q\ge {p-1}$ yields a solution $\bar{u}_h$ that approximates optimally the true solution *u*, presenting convergence order *p* for the error measured in the $H^1$ semi-norm when the mesh size $h\rightarrow 0$. In [66], the authors also observed, through numerical experiments, that a projection degree $q>p-1$ yields optimal convergence order also respect to the $L^2$ norm of the error (rate $p+1$).

#### Remark 1

The non-polynomial nature of the quantities ${\varvec{K}}$, *f*, and *g* may derive from an additional mapping that further deforms the domain $\Omega _0$ (see, e.g., [67]). A numerical example addressing this case is presented in Sect. 5.2.1 (the multi-perforated quarter of annulus). On the contrary, these quantities might be low-order polynomials (even zero-order polynomials) by construction and it is therefore not necessary to project them into polynomial spaces.

In [66], the projections (13) are performed patch-wise. Nevertheless, the same error estimates hold in the case they are carried out in an element-wise way, that is the case of this work. This results in polynomial approximations that are element-wise discontinuous. Thus, for each element $Q\in {\mathcal {T}}_h(\Omega )$ we introduce a local $L^2$-projector:

14 $$\begin{aligned} \Pi ^{h}_{Q}:{L}^2(Q) \rightarrow \mathbb {Q}_{q,\,q,\,q}(Q),\quad \forall Q\in {\mathcal {T}}_h(\Omega ), \end{aligned}$$

where $\mathbb {Q}_{q_1,q_2,\dots ,q_{m}}$ denotes the space of tensor-product polynomials with degrees $(q_1,q_2,\dots ,q_{m})$ along the *m* parametric directions.

By employing a tensor-product Bernstein basis, the projected quantities $\bar{{\varvec{K}}}$, $\bar{f}$, and $\bar{g}$ restricted to element *Q* can be expressed as:

15 $$\begin{aligned} \begin{aligned} \bar{{\varvec{K}}}|_{Q} = \sum ^{\left( q+1\right) ^3}_{k=1} B^{\mathbf {q}}_k\,\bar{K}^{(Q)}_k,\\ \bar{f}|_{Q} = \sum ^{\left( q+1\right) ^3}_{k=1} B^{\mathbf {q}}_k\,\bar{f}^{(Q)}_k,\\ \bar{g}|_{Q} = \sum ^{\left( q+1\right) ^3}_{k=1} B^{\mathbf {q}}_k\,\bar{g}^{(Q)}_k, \end{aligned} \end{aligned}$$

where $\bar{K}^{(Q)}_k\in \mathbb {R}^{3\times {3}}$, $\bar{f}^{(Q)}_k\in \mathbb {R}$, and $\bar{g}^{(Q)}_k\in \mathbb {R}$ are the projection coefficients, and $B^{\mathbf {q}}_k$ are tensor-product Bernstein polynomials defined over *Q* and with degrees $\mathbf {q}=(q,q,q)$ such that

16 $$\begin{aligned} \mathbb {Q}_{q,\,q,\,q}(Q) = \text {span}\{B^{\mathbf {q}}_{k}\,\ k=1,\dots ,\left( q + 1\right) ^3\}. \end{aligned}$$

We refer the interested reader to the Sect. 1 of Appendix A for a discussion about tensor-product Bernstein polynomials.

**Fig. 2:**
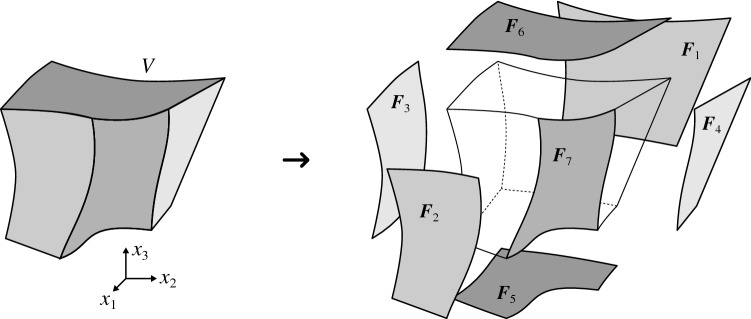
Boundary representation of a volumetric domain *V*

### Operators assembly through lookup tables

In what follows, we detail the assembly of the elemental stiffness matrix and the right-hand-side vector associated to the operators (12). Thus, plugging the projections (15) into (12), a single entry of the elemental matrix and vector can be computed as:

17 $$\begin{aligned} \mathsf {A}_{ij}^{(Q)}&= \sum _{k=1}^{(q+1)^3} \bar{K}^{(Q)}_k: \int _{Q\cap \Omega } B^{\mathbf {q}}_k \big (\nabla {N_i} \otimes \nabla {N_j}\big ) \,{\text {d}}Q, \\ \mathsf{b}_{i}^{(Q)} &= \sum_{k=1}^{(q+1)^3} \bar{f}^{(Q)}_k \int_{Q\cap\Omega} B^{\mathbf{q}}_k N_i \,{\text{d}}Q + \sum_{k=1}^{(q+1)^3} \bar{g}^{(Q)}_k \int_{Q\cap\Gamma_N} B^{\mathbf{q}}_k N_i \,{\text{d}} \Gamma, \end{aligned}$$

where $N_i,N_j\in \mathbb {V}(\Omega )$ are test and trial basis functions, respectively. In the expressions above it is easy to realize that all the integrands restricted to a single element *Q* are polynomials:

18a $$\begin{aligned}&B^{\mathbf {q}}_k \big (\nabla {N_i}\otimes \nabla {N_j}\big )|_{Q} \in \mathbb {Q}_{2 p +q,\,2 p +q,\,2 p +q}(Q)\,, \end{aligned}$$

18b $$\begin{aligned}&B^{\mathbf {q}}_k N_i|_{Q} \in \mathbb {Q}_{p +q,\,p +q,\,p +q}(Q)\,. \end{aligned}$$

Notice also that the functions $N_i$, $N_j$, and $B^{\mathbf {q}}_k$ are naturally defined over the full support of each element *Q*, and not only over its active part $Q\cap \Omega$.

Finally, by exploiting their polynomial nature, the element integrals in (17) can be computed as:

19a $$\begin{aligned} \int _{Q\cap \Omega } B^{\mathbf {q}}_k\big (\nabla {N_i}&\otimes \nabla {N_j}\big ) \,{\text {d}}Q \nonumber \\&= \sum ^{\left( 2 p + q + 1\right) ^3}_{\alpha =1} \mathbf {\mathsf {K}}^{(Q)}_{i,j,k,\alpha } \int _{Q\cap \Omega } B^{\mathbf {r}}_{\alpha } {\text {d}}Q \end{aligned}$$

19b $$\begin{aligned} \int _{Q\cap \Omega } B^{\mathbf {q}}_k N_i \,{\text {d}}Q&= \sum ^{\left( p + q + 1\right) ^3}_{\beta =1} \mathsf {F}^{(Q)}_{i,k,\beta } \int _{Q\cap \Omega } B^{\mathbf {s}}_{\beta } {\text {d}}Q \end{aligned}$$

19c $$\int_{Q\cap\Gamma_N} B^{\mathbf{q}}_k N_i \,{\text{d}} Q = \sum^{\left(p + q + 1\right)^3}_{\beta=1} \mathsf{G}^{(Q)}_{i,k,\beta} \int_{Q\cap\Gamma_N} B^{\mathbf{s}}_{\beta} {\text{d}} \Gamma$$

where $B^{\mathbf {r}}_{\alpha }$ and $B^{\mathbf {s}}_{\beta }$ are tensor-product Bernstein polynomials with degrees $\mathbf {r}=(2 p + q,\,2 p + q,\,2 p + q)$ and $\mathbf {s}=(p + q,\,p + q,\,p + q)$. $\mathbf {\mathsf {K}}^{(Q)}_{i,j,k,\alpha }\in \mathbb {R}^{3\times 3}$ and $\mathsf {F}^{(Q)}_{i,k,\beta },\,\mathsf {G}^{(Q)}_{i,k,\beta }\in \mathbb {R}$ are element dependent constant coefficients that can be calculated by means of the Bézier extraction operators [68–70] associated to the spline space $\mathbb {V}_h(\Omega )$.

Then, the assembly of the operators (17) reduces to the computation of the coefficients $\mathbf {\mathsf {K}}^{(Q)}_{i,j,k,\alpha }$, $\mathsf {F}^{(Q)}_{i,k,\beta }$, and $\mathsf {G}^{(Q)}_{i,k,\beta }$, as well as the integrals^1^:

20 $$\begin{aligned} \mathsf {I}^{3D }_{Q,\alpha } = \int _{Q\cap \Omega } B^{\mathbf {r}}_{\alpha } {\text {d}}Q,\quad \mathsf{I}^{2\textup{D}}_{Q,\beta} = \int_{Q\cap\Gamma_N} B^{\mathbf{s}}_{\beta} {\text{d}} \Gamma. \end{aligned}$$

Thus, the integrals $\mathsf {I}^{3D }_{Q,\alpha }$ and $\mathsf {I}^{2D }_{Q,\beta }$ can be precomputed for every element *Q* and stored in lookup tables, that will be accessed along the assembly process to create the elemental operators, in a similar way as proposed in [66].

Nevertheless, as discussed in Sect. 1, the computation of the integrals (20) is a challenging task. In the case of non-cut elements, their evaluation is straightforward: It can be precomputed analytically for a single unit cube and subsequently adapted to every non-cut element’s domain through simple transformations (translations and scalings). But in the case of cut elements the evaluation of the integrals $\mathsf {I}^{3D }_{Q,\alpha }$ and $\mathsf {I}^{2D }_{Q,\beta }$ is far from simple. For that purpose, in Sect. 4 we propose a quadrature-free approach for the common case in which the active part of elements ($Q\cap \Omega$) can be defined through a B-Rep, discussed in Sect. 3.

## Geometric modeling via boundary representation

In this section, we introduce the notation and some basic concepts about splines and geometric modeling. Hence, we provide a mathematical way of describing the active part of the cut elements $Q\cap \Omega$, discussed in the previous section, by means of B-Rep representations. This constitutes the basis for the integration method presented in Sect. 4.

**Fig. 3:**
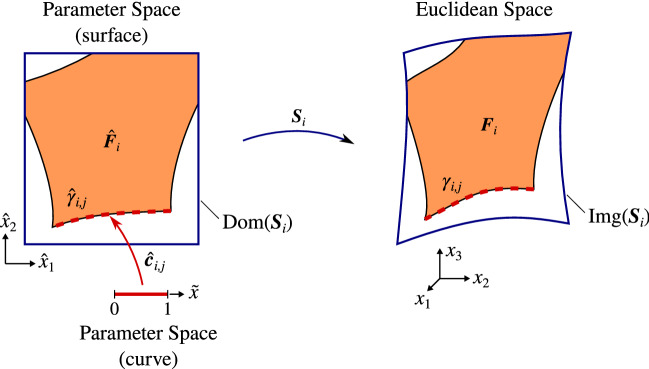
Description of the involved geometrical entities in the definition of trimmed parametric surfaces

### Spline representation

Splines are considered a *de facto* standard in Computer-Aided Design and have been extensively studied in the literature, see for instance [71–73]. Among the different representation techniques available, in this work we focus on the use of polynomial mappings, and more specifically, B-spline and Bézier curves and surfaces. A B-spline or Bézier curve ${\varvec{c}}$ can be expressed in the form:

21 $$\begin{aligned} {\varvec{c}}:[0,1] \rightarrow \mathbb {R}^d,~~ \tilde{x}\mapsto \varvec{c}(\tilde{x}) = \sum _{i=1}^{n} N^{p}_i(\tilde{x}) \varvec{P}_{i}, \end{aligned}$$

where $N^{p}_i$ are univariate basis functions, either B-splines or Bernstein polynomials, of degree *p*, and $\varvec{P}_i\in \mathbb {R}^d$ are their associated control points, where *d* is the number of spatial dimensions. In Appendix A we provide further details about Bernstein polynomials (Appendix A.1 and A.2) and Bézier geometries (Appendix A.3), that are extensively used in this work. For an in-depth discussion about B-splines, we refer the interested reader to the existing literature [71–73].

Using tensor-product combinations of those basis functions, B-spline and Bézier surfaces ${\varvec{S}}$ can be constructed as:

22 $$\begin{aligned} \begin{aligned} {\varvec{S}}:~~[0,1]^{2}&\rightarrow \mathbb {R}^d,\\ (\hat{x}_1,\hat{x}_2)&\mapsto {\varvec{S}}(\hat{x}_1,\hat{x}_2)\sum _{i=1}^{n_1}\sum _{j=1}^{n_2} N^{p_1}_i(\hat{x}_1)N^{p_2}_j(\hat{x}_2) \varvec{P}_{i,j}, \end{aligned} \end{aligned}$$

where $N^{p_1}_i$ and $N^{p_2}_j$ are univariate B-spline or Bernstein basis functions of degrees $p_1$ and $p_2$, respectively, and $\varvec{P}_{i,j}\in \mathbb {R}^d$ are the associated control points. For the sake of simplicity, we assumed the parametric domains of the mappings (21) and (22), ${\text {Dom}}({\varvec{c}})$ and ${\text {Dom}}({\varvec{S}})$, to be [0, 1] and $[0,1]^2$, respectively.

### Trimmed surfaces and boundary representations

Simple spline mappings (21) and (22) cannot represent complex real-world geometries. Instead, the multitude of these geometric objects are usually combined for such a purpose. More specifically, Boolean operations (namely, unions, differences, and/or intersections) of several geometrical entities are commonly adopted in Computer-Aided Design [71]. By means of these operations, volumetric geometries are often represented in an implicit way: the volume enclosed by a set of, possibly trimmed, boundaries surfaces. This paradigm, known as Boundary Representation (B-Rep) [74, 75] and extensively used in industrial modeling tools, is considered throughout this work.

As illustrated in Fig. 2, we consider a domain $V\subset \mathbb {R}^3$, non-simply connected in general, whose boundary $\partial V$ is defined by a set of connected faces ${F_i},~i=1,\dots ,n_F$, such as:

23 $$\begin{aligned} \partial V=\cup _{i=1}^{n_F}{F_i}. \end{aligned}$$

The domain *V* may correspond to the active part of the cut elements $Q\cap \Omega$ discussed in Sect. 2.1.

**Fig. 4:**
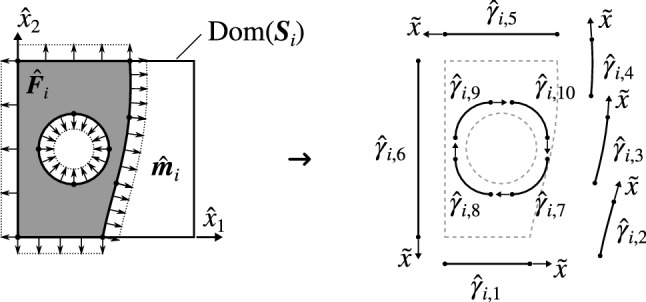
Boundary representation of trimmed faces. External boundaries follow a counter-clockwise orientation while the internal ones are clockwise oriented

We consider the faces ${F_i}$ to be defined as trimmed B-spline or Bézier surfaces that are piecewise smooth. Every trimmed face ${F_i}$ is composed of two elements: an underlying spline surface mapping ${{\varvec{S}}_i}$ of the form (22), and a group of connected curvilinear segments ${\hat{\gamma }_{i,j}}\subset {\text {Dom}}({{\varvec{S}}_i}),~j=1,\dots ,n_{c,i}$, that delimit the active region of ${\text {Dom}}({{\varvec{S}}_i})$ (see Figs. 3 and 4). We denote this active region as ${\hat{F}_i}\subset {\text {Dom}}({{\varvec{S}}_i})$.

Each segment ${\hat{\gamma }_{i,j}}$ is the image of a spline curve mapping ${\hat{{\varvec{c}}}_{i,j}}:[0,1] \rightarrow {\hat{\gamma }_{i,j}}$ of the form (21). Thus, the boundary of the active region ${\hat{F}_i}$ is:

24 $$\begin{aligned} \begin{aligned} \partial {\hat{F}_i}&= \cup _{j=1}^{n_{c,i}} {\hat{\gamma }_{i,j}},\\ {\hat{\gamma }_{i,j}}&=\{ {\hat{{{\varvec{x}}}}}\in \mathbb {R}^2\ \vert \ {\tilde{x}}\in [0,1]\,\ {\hat{{{\varvec{x}}}}}={\hat{{\varvec{c}}}_{i,j}}({\tilde{x}}) \}, \end{aligned} \end{aligned}$$

therefore, we can define ${F_i}$ as:

25 $$\begin{aligned} {F_i}=\{ {{\varvec{x}}}\in \mathbb {R}^3 \ \vert \ {\hat{{{\varvec{x}}}}}\in {\hat{F}_i}\,\ {{\varvec{x}}}={{\varvec{S}}_i}({\hat{{{\varvec{x}}}}}) \}. \end{aligned}$$

We again refer to Fig. 3 where all the introduced quantities are depicted for an illustrative example.

#### Remark 2

**Fig. 5:**
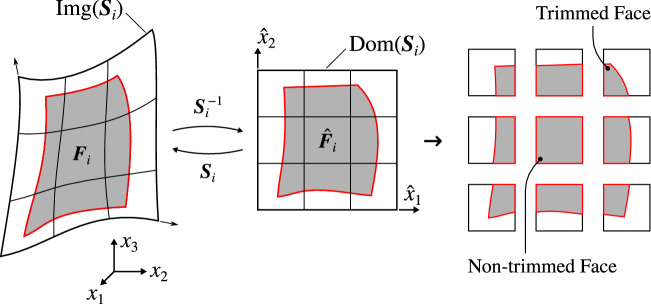
Split of a trimmed B-spline surface into trimmed Bézier surfaces

To work exclusively with pure polynomial representations, instead of (rational) piecewise polynomials, in this work we only consider non-rational Bézier curves and surfaces. Using only Béziers does not constitute any limitation: By refining at its internal knots, any face ${F_i}$, defined by means of B-spline curves and surfaces, can be easily split into a set of trimmed Bézier faces, whose underlying curves and surfaces are Béziers (see Fig. 5). On the other hand, the exclusive use of non-rational polynomials may be a limiting factor as it turns impossible the creation of exact conic curves and surfaces.

This limitation can be circumvented in the case of the resolution of elliptic PDEs using immersed IGA. As discussed in [67], in those cases it is possible to approximate the geometry of the cut elements $Q\cap \Omega \ \forall Q\in {\mathcal {T}\,}^{\Gamma }_h(\Omega )$ by means of Bézier curves and surfaces of degree *p*, the same as the solution’s discretization, and still preserve optimal approximation properties.

## Quadrature-free integration of polynomials over B-Reps

In this section, we deal with the integration of polynomials over a domain *V* whose bounding faces ${F_i}$ are represented as trimmed Bézier surfaces, as described in the previous section. More specifically, we seek to compute the integral:

26 $$\begin{aligned} I^{3D } = \iiint _{V} a \,{\text {d}}V\,, \end{aligned}$$

where $a:V\rightarrow \mathbb {R}$ is a polynomial function. This addresses the computation of the integrals $\mathsf {I}^{3D }_{Q,\alpha }$ over cut elements $Q\cap \Omega$ as described in (20).

The approach presented in this section consists in the successive application of the divergence theorem, as similarly done, for instance, in [45, 51, 54, 76]. Let us first recall here the classical divergence theorem, also known as Gauss-Ostrogradsky’s theorem.

### Theorem 1

Let *V* be a subset of $\mathbb {R}^3$ which is compact and has a piecewise smooth boundary $\partial V$. Let ${\varvec{A}}$ be a three-dimensional vector field, such that ${\varvec{A}}:V\rightarrow \mathbb {R}^3$ and ${\varvec{A}}\in [C^1(V)]^3$, then:

27 $$\begin{aligned} \iiint _{V} \nabla \cdot {\varvec{A}}\, {\text {d}}V = \iint _{\partial V} {\varvec{A}}\cdot {{\varvec{n}}}\, {\text {d}}S\,, \end{aligned}$$

where $\nabla \cdot$ is the divergence operator and ${{\varvec{n}}}:\partial V\rightarrow \mathbb {R}^3$ is the outward pointing unit normal on the boundary $\partial V$.

By applying the divergence theorem, the three-dimensional integral (26) is transformed into, first, surface, and then line integrals that can be evaluated analytically with machine precision accuracy. This is possible in the present context due to the polynomial nature of the successive integrands which ease the formation of the antiderivatives involved in the integration process.

### From volume integral to surface integrals

To apply the divergence theorem, let us first rewrite the initial integral (26) in the same form as the one in (27):

28 $$\begin{aligned} I^{3D } = \iiint _{V} \nabla \cdot {\varvec{A}}\,{\text {d}}V\,. \end{aligned}$$

The vector field ${\varvec{A}}:V\rightarrow \mathbb {R}^3$ can be expressed as:

29 $$\begin{aligned} {\varvec{A}}({{\varvec{x}}}) = A_1({{\varvec{x}}}){\varvec{e}}_{1} + A_2({{\varvec{x}}}){\varvec{e}}_{2} + A_3({{\varvec{x}}}){\varvec{e}}_{3}, \end{aligned}$$

with ${\varvec{e}}_{i}$ as the Cartesian unit vectors and $Q_i:V\rightarrow \mathbb {R}$ as the antiderivatives of *a*, computed by:

30 $$\begin{aligned} \begin{aligned} A_1(x_1,x_2,x_3)&= \alpha _1\int _{0}^{x_1} a(\sigma ,x_2,x_3){\text {d}}{\sigma } + \beta _1,\\ A_2(x_1,x_2,x_3)&= \alpha _2\int _{0}^{x_2} a(x_1,\sigma ,x_3){\text {d}}{\sigma } + \beta _2,\\ A_3(x_1,x_2,x_3)&= \alpha _3\int _{0}^{x_3} a(x_1,x_2,\sigma ){\text {d}}{\sigma } + \beta _3. \end{aligned} \end{aligned}$$

Here $\alpha _1$, $\alpha _2$, $\alpha _3$, $\beta _1$, $\beta _2$, and $\beta _3$ are real constants, such that $\alpha _1+\alpha _2+\alpha _3=1$. Since *a* is a polynomial function, the computation of the antiderivatives in (30) is straightforward [see Appendix A, Eq. (59)]. Furthermore, due to this polynomial nature, the continuity requirements of the divergence theorem are granted for the vector field ${\varvec{A}}$.

Applying the divergence theorem to (28) we obtain:

31 $$\begin{aligned} I^{3D } = \iint _{\partial V} {\varvec{A}}\cdot {{\varvec{n}}}\,{\text {d}}S\,, \end{aligned}$$

where we recall that ${{\varvec{n}}}:\partial V\rightarrow \mathbb {R}^3$ is the outward pointing unit normal on the boundary $\partial V$. Recalling the definition of the boundary $\partial V$ in (23), the integral (31) can be split as:

32 $$\begin{aligned} I^{3D } = {\sum _{i=1}^{n_F}}\,I_i^{2D } = {\sum _{i=1}^{n_F}}\iint _{{F_i}} {{\varvec{A}}\cdot {{\varvec{n}}}_i} \,{\text {d}}S_i\,, \end{aligned}$$

where ${{\varvec{n}}}_i$ are the outward pointing unit normals of the surfaces ${{\varvec{S}}_i}$, $i=1,\dots ,n_F$. Exploiting the parametric representation of the surfaces ${{\varvec{S}}_i}$, these unit normal vector fields can be expressed as:

33 $$\begin{aligned} {{\varvec{n}}}_i: \text {Img}({{\varvec{S}}_i}) \rightarrow \mathbb {R}^3,~ {{\varvec{x}}}\mapsto \bigg ( \frac{{{\varvec{N}}}_i}{\Vert {{\varvec{N}}}_i\Vert } \circ {{\varvec{S}}_i}^{-1} \bigg )({{\varvec{x}}}), \end{aligned}$$

where the normal vectors ${{\varvec{N}}}_i$ are computed as:

34 $$\begin{aligned} {{\varvec{N}}}_i: {\text {Dom}}({{\varvec{S}}_i}) \rightarrow \mathbb {R}^3,~ {\hat{{{\varvec{x}}}}}\mapsto \bigg ( \frac{\partial {{\varvec{S}}_i}}{\partial \hat{x}_1} \times \frac{\partial {{\varvec{S}}_i}}{\partial \hat{x}_2} \bigg )({\hat{{{\varvec{x}}}}}). \end{aligned}$$

In (34) we assumed that the surface parameterization is oriented such that the cross-product ${{\varvec{N}}}_i$ points out of *V*. Plugging (33) into the expression of the surface integrals $I^{2D }$ in (32), they become:

35 $$\begin{aligned} I_i^{2D } = \iint _{{F_i}} {\varvec{A}}\cdot \left( \frac{{{\varvec{N}}}_i}{\Vert {{\varvec{N}}}_i\Vert } \circ {{\varvec{S}}_i}^{-1}\right) \,{\text {d}}S_i\,, \end{aligned}$$

for $i = 1,\dots {},n_F$. And pulling back these integrals to the parametric domain of ${{\varvec{S}}_i}$, we obtain:

36 $$\begin{aligned} I_i^{2D } = \iint _{{\hat{F}_i}} {\hat{r}_i}\,{{\text {d}}\hat{{{\varvec{x}}}}}, \end{aligned}$$

where the integrands ${\hat{r}_i}$ are defined as:

37 $$\begin{aligned} \begin{aligned} {\hat{r}_i}: {\text {Dom}}({{\varvec{S}}_i})&\rightarrow \mathbb {R},\\ {\hat{{{\varvec{x}}}}}&\mapsto {\hat{r}_i}({\hat{{{\varvec{x}}}}})= \big ({\varvec{A}}\circ {{\varvec{S}}_i}\big )({\hat{{{\varvec{x}}}}}) \cdot {{\varvec{N}}}_i({\hat{{{\varvec{x}}}}}). \end{aligned} \end{aligned}$$

Interestingly, the normalization and the inversion involved in the definition of the unit normal vectors (33) vanish after the pull-back, as observed in [46], for instance. Furthermore, as the surface ${{\varvec{S}}_i}$ is assumed to be polynomial, then the composition ${\varvec{A}}\circ {{\varvec{S}}_i}$ is also a polynomial bivariate, but with a higher degree. Additionally, the non-normalized normal vector field ${{\varvec{N}}}_i$ is also a polynomial since it is computed as the product of polynomial terms (the partial derivatives of ${{\varvec{S}}_i}$ are polynomials). Finally, the scalar product of two polynomial vector fields, ${\varvec{A}}\circ {{\varvec{S}}_i}$ and ${{\varvec{N}}}_i$, is a polynomial scalar field. Consequently, ${\hat{r}_i}$ is a polynomial. In the case of Bernstein polynomials we refer the interested reader to Appendix A: see Eq. (75) for the details of the composition ${\varvec{A}}\circ {{\varvec{S}}_i}$ between a trivariate and a surface; and Eq. (71) for the multiplications of multivariate polynomials involved in the cross and scalar products of Eqs. (34) and (37), respectively.

#### Remark 3

The integrals $I_i^{2D }$ in (36) are equivalent to the boundary integrals $\mathsf {I}^{2D }_{Q,\beta }$ depicted in (20) and required for the assembly of boundary conditions in immersed methods (see Sect. 2).

#### Remark 4

In the case of non-trimmed Bézier surfaces, like the one depicted in Fig. 5, the integrals (36) can be easily evaluated analytically using Eq. (69).

#### Remark 5

In some situations the normal fields ${{\varvec{n}}}_i$ of the surfaces ${{\varvec{S}}_i}$ may be aligned with one of three the Cartesian axes. This occurs quite often in the case of immersed methods for solving PDEs, presented in Sect. 2, in which the integration domains *V* correspond to the cut elements $Q\cap \Omega \ \forall Q\in {\mathcal {T}}_h(\Omega )$ of the grid embedded in a B-Rep geometry. In that particular situation many faces ${F_i}$ will be planar trimmed surfaces parallel to the Cartesian axes. For those cases, a wise choice of the coefficients $\alpha _1$, $\alpha _2$, and $\alpha _3$ in the antiderivatives (30) will make the scalar product ${\varvec{A}}\cdot {{\varvec{n}}}_i$ vanish, minimizing the number of two-dimensional integrals to be computed. For instance, in the case of a face ${F_i}$ that is perpendicular to the *z* Cartesian axis, choosing $\alpha _3=0$ will make the term ${\varvec{A}}\cdot {{\varvec{n}}}_i$ vanish. Nevertheless, for a given domain *V* the coefficients $\alpha _1$, $\alpha _2$, and $\alpha _3$ must be set once and for all, and cannot be independently chosen for every face ${F_i}$ of *V*. Thus, an optimal strategy may be to set $\alpha _1$, $\alpha _2$, and $\alpha _3$ independently for every *V* such that the largest number of surface integrals vanish for that specific domain.

### Evaluating the surface boundary integrals

Applying again the divergence theorem (27), we can transform the two-dimensional integrals $I_i^{2D }$ in (36) into line integrals as:

38 $$\begin{aligned} I_i^{2D } = \int _{\partial {\hat{F}_i}} {\hat{{\varvec{R}}}_i}\cdot \hat{{{\varvec{m}}}}_i \,{\text {d}}\ell _i , \end{aligned}$$

where $\hat{{{\varvec{m}}}}_i:\partial {\hat{F}_i}\rightarrow \mathbb {R}^2$ is the outward pointing unit normal on the boundary $\partial {\hat{F}_i}$. The vector field ${\hat{{\varvec{R}}}_i}:{\text {Dom}}({{\varvec{S}}_i})\rightarrow \mathbb {R}^2$ is defined such that ${\hat{r}_i}= {\hat{\nabla }}\cdot {\hat{{\varvec{R}}}_i}$, as for instance:

39 $$\begin{aligned} \begin{aligned} {\hat{{\varvec{R}}}_i}({\hat{x}}_1,{\hat{x}}_1)&= \bigg (\delta _1\int _{0}^{{\hat{x}}_1} {\hat{r}_i}(\sigma ,{\hat{x}}_2) \,{\text {d}}\sigma + \epsilon _1 \bigg ){\varvec{e}}_{1}\\& \quad + \bigg (\delta _2\int _{0}^{{\hat{x}}_2} {\hat{r}_i}({\hat{x}}_1,\sigma ) \,{\text {d}}\sigma + \epsilon _2 \bigg ){\varvec{e}}_{2}, \end{aligned} \end{aligned}$$

and $\delta _1$, $\delta _2$, $\epsilon _1$, and $\epsilon _2$ are real constants, such that $\delta _1+\delta _2=1$.

Splitting the boundary $\partial {\hat{F}_i}$ according to (24) we obtain:

40 $$\begin{aligned} I_i^{2D } = {\sum _{j=1}^{n_{c,i}}}I_{i,j}^{1D } = {\sum _{j=1}^{n_{c,i}}}\int _{{\hat{\gamma }_{i,j}}} {\hat{{\varvec{R}}}_i}\cdot \hat{{{\varvec{m}}}}_{i,j} \,{\text {d}}\ell _{i,j}, \end{aligned}$$

where $\hat{{{\varvec{m}}}}_{i,j}:\text {Img}({\hat{{\varvec{c}}}_{i,j}})\rightarrow \mathbb {R}^2$ are the outward pointing unit normals of the curves ${\hat{{\varvec{c}}}_{i,j}}$, $i=1,\dots ,n_{c,i}$. Exploiting the parametric representation of the curves ${\hat{{\varvec{c}}}_{i,j}}$, these unit normal vector fields can be expressed as,

41 $$\begin{aligned} \hat{{{\varvec{m}}}}_{i,j}: \text {Img}({\hat{{\varvec{c}}}_{i,j}}) \rightarrow \mathbb {R}^2,~ {\hat{{{\varvec{x}}}}}\mapsto \bigg ( \frac{\hat{{{\varvec{M}}}}_{i,j}}{\Vert \hat{{{\varvec{M}}}}_{i,j}\Vert } \circ {\hat{{\varvec{c}}}_{i,j}}^{-1} \bigg )({\hat{{{\varvec{x}}}}}), \end{aligned}$$

where the normal vectors $\hat{{{\varvec{M}}}}_{i,j}$ are computed as:

42 $$\begin{aligned} \hat{{{\varvec{M}}}}_{i,j}: {\text {Dom}}({\hat{{\varvec{c}}}_{i,j}}) \rightarrow \mathbb {R}^2,~ {\tilde{x}}\mapsto \frac{{\text {d}}{\hat{{\varvec{c}}}_{i,j}}}{{\text {d}}{\tilde{x}}}({\tilde{x}}) \times {\varvec{e}}_{3} . \end{aligned}$$

In the previous expression, we assume that the curves ${\hat{{\varvec{c}}}_{i,j}}$ are oriented such as the external boundaries of ${\hat{F}_i}$ present a counter-clockwise orientation, while the internal ones are clockwise oriented (see Fig. 4).

Plugging (41) into the expression of the line integrals $I^{1D }$ involved in (40), they become:

43 $$\begin{aligned} I_{i,j}^{1D } = \int _{{\hat{\gamma }_{i,j}}} {\hat{{\varvec{R}}}_i}\cdot \bigg (\frac{\hat{{{\varvec{M}}}}_{i,j}}{\Vert \hat{{{\varvec{M}}}}_{i,j}\Vert } \circ {\hat{{\varvec{c}}}_{i,j}}^{-1}\bigg ) \,{\text {d}}\ell _{i,j}\,. \end{aligned}$$

Finally, pulling back these integrals to the parametric domain of the underlying curves ${\hat{{\varvec{c}}}_{i,j}}$, we obtain:

44 $$\begin{aligned} I_{i,j}^{1D } = \int _{0}^{1} \big ({\hat{{\varvec{R}}}_i}\circ {\hat{{\varvec{c}}}_{i,j}}\big ) \cdot \hat{{{\varvec{M}}}}_{i,j} \,{{\text {d}}\tilde{x}}, \end{aligned}$$

where, as for the two-dimensional case, the normalization and the inversion involved in the definition of the unit normal vectors (41) vanish after the pull-back. We gather all the integrand terms together as:

45 $$\begin{aligned} I_{i,j}^{1D } = \int _{0}^{1} \tilde{t}_{i,j} \,{{\text {d}}\tilde{x}}, \end{aligned}$$

where

46 $$\begin{aligned} \begin{aligned} \tilde{t}_{i,j}:&{\text {Dom}}({\hat{{\varvec{c}}}_{i,j}})=[0,1] \rightarrow \mathbb {R},\\&{\tilde{x}}\mapsto \tilde{t}({\tilde{x}})= \big ({\hat{{\varvec{R}}}_i}\circ {\hat{{\varvec{c}}}_{i,j}}\big )({\tilde{x}}) \cdot \hat{{{\varvec{M}}}}_{i,j}({\tilde{x}}). \end{aligned} \end{aligned}$$

As the curve ${\hat{{\varvec{c}}}_{i,j}}$ is a Bézier, the composition ${\hat{{\varvec{R}}}_i}\circ {\hat{{\varvec{c}}}_{i,j}}$ is a higher degree univariate polynomial. Additionally, the non-normalized normal vector field $\hat{{{\varvec{M}}}}_{i,j}$ is also a polynomial since it is computed from Bézier derivatives. Finally, the scalar product of two polynomial vector fields, ${\hat{{\varvec{R}}}_i}\circ {\hat{{\varvec{c}}}_{i,j}}$ and $\hat{{{\varvec{M}}}}_{i,j}$, is a polynomial scalar field. Consequently, $\tilde{t}_{i,j}$ is a polynomial. Therefore, the integrals (45) can be easily evaluated in an analytic way, with machine precision accuracy, without the need for quadrature schemes.

Further details for the case of Bernstein polynomials are provided in Appendix A: the composition ${\hat{{\varvec{R}}}_i}\circ {\hat{{\varvec{c}}}_{i,j}}$ between a bivariate and a curve is detailed in Eq. (75); the derivative involved in (42) is easily determined by computing the derivatives of the Bernstein basis functions as described in (58); the scalar product in (46) can be evaluated by computing the product of the individual components (Eq. (66)) and then summing the resulting expressions (Eq. (65)); finally, the 1D integrals (45) can be analytically determined using the expression (63).

#### Remark 6

The Remark 5 is extensible to the line integrals detailed above. In some situations (see for instance Fig. 4), some boundaries ${\hat{\gamma }_{i,j}}$ may be aligned with the Cartesian axes. In those cases, the constants $\delta _1$ and $\delta _2$ arising in the antiderivatives (39) can be chosen such as the product ${\hat{{\varvec{R}}}_i}\cdot \hat{{{\varvec{m}}}}_{i,j}$ vanishes in some of those boundaries. These constants can be chosen independently for every face integral $I_{i}^{2D }$ such as the number of 1D integrals to be evaluated is minimized.

**Fig. 6:**
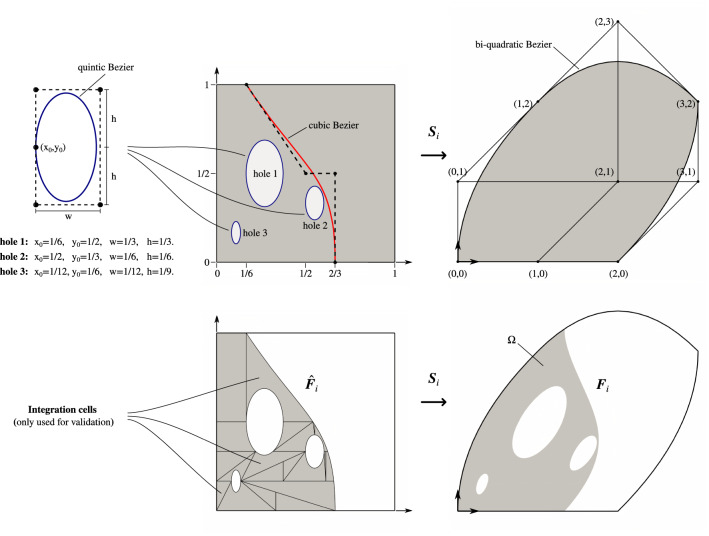
The two-dimensional trimmed geometry for the validation of the quadrature-free integration procedure

### Polynomial degree

The reader may have noticed that due to the involved compositions, ${\varvec{A}}\circ {{\varvec{S}}_i}$ and ${\hat{{\varvec{R}}}_i}\circ {\hat{{\varvec{c}}}_{i,j}}$, as well as the products of Bézier curves and surfaces, the resulting polynomial term $\tilde{t}_{i,j}$ can potentially present a very high degree. In this section, we detail the computation of this degree, as well as the order of other terms involved in the intermediate steps.

For the sake of simplicity, hereinafter we assume that the polynomial *a* to integrate, as well as the Bézier mappings ${{\varvec{S}}_i}$ and ${\hat{{\varvec{c}}}_{i,j}}$, have constant degrees along all their parametric directions and for all their components:

47 $$\begin{aligned} a \in \mathbb {Q}_{r,r,r};~ {{\varvec{S}}_i}\in \mathbb {Q}_{s,s}\times \mathbb {Q}_{s,s}\times \mathbb {Q}_{s,s};~ {\hat{{\varvec{c}}}_{i,j}}\in \mathbb {Q}_c\times \mathbb {Q}_c, \end{aligned}$$

with $r\ge 0$, $s>0$, and $c>0$, and where the polynomial spaces $\mathbb {Q}$ follow the notation introduced in Sect. 2.2. According to the definitions (34) and (42) it is straightforward to obtain the degrees of the fields ${{\varvec{N}}}_i$ and ${\hat{{{\varvec{M}}}}}_{i,j}$ as:

48 $$\begin{aligned} \begin{aligned} {{\varvec{N}}}_i&\in \mathbb {Q}_{2s-1,\,2s-1}\times \mathbb {Q}_{2s-1,\,2s-1}\times \mathbb {Q}_{2s-1,\,2s-1},\\ {\hat{{{\varvec{M}}}}}_{i,j}&\in \mathbb {Q}_{c-1}\times \mathbb {Q}_{c-1}, \end{aligned} \end{aligned}$$

and using (30), the order of ${\varvec{A}}$ is computed as:

49 $$\begin{aligned} {\varvec{A}}\in \mathbb {Q}_{r+1,\,r,\,r}\times \mathbb {Q}_{r,\,r+1,\,r}\times \mathbb {Q}_{r,\,r,\,r+1}. \end{aligned}$$

Thus, the degrees of ${\varvec{A}}\circ {{\varvec{S}}_i}$ and ${\hat{r}_i}$ [recall Eq. (37)] are:

50 $$\begin{aligned} \begin{aligned}&{\varvec{A}}\circ {{\varvec{S}}_i}\in \mathbb {Q}_{t,\,t}\times \mathbb {Q}_{t,\,t}\times \mathbb {Q}_{t,\,t},\quad {}t= 2\left( 3 r + 1\right) ,\\&{\hat{r}_i}\in \mathbb {Q}_{3s\left( r+1\right) -1,\,3s\left( r+1\right) -1}. \end{aligned} \end{aligned}$$

**Fig. 7:**
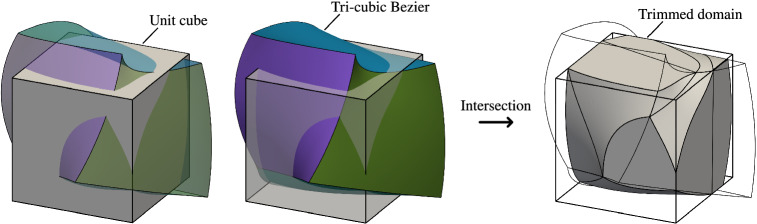
The three-dimensional trimmed geometry for the validation of the quadrature-free integration procedure

Analogously to the case of ${\varvec{A}}$, the degree of ${\hat{{\varvec{R}}}_i}$ (Eq. 39), and its composition ${\hat{{\varvec{R}}}_i}\circ {\hat{{\varvec{c}}}_{i,j}}$, are simply computed as:

51 $$\begin{aligned} \begin{aligned}&{\hat{{\varvec{R}}}_i}\in \mathbb {Q}_{3s\left( r+1\right) ,\,3s\left( r+1\right) -1}\times \mathbb {Q}_{3s\left( r+1\right) -1,\,3s\left( r+1\right) },\\&{\hat{{\varvec{R}}}_i}\circ {\hat{{\varvec{c}}}_{i,j}}\in \mathbb {Q}_{6sc\left( r+1\right) -c}\times \mathbb {Q}_{6sc\left( r+1\right) -c}. \end{aligned} \end{aligned}$$

Finally, the polynomial term $\tilde{t}_{i,j}$ presents a degree:

52 $$\begin{aligned} \tilde{t}_{i,j} \in \mathbb {Q}_{6sc\left( r+1\right) -1}. \end{aligned}$$

The degree of $\tilde{t}_{i,j}$ can be potentially very high what may induce numerical instabilities. Nevertheless, in the examples of Sect. 5.2.2 very high order polynomials were involved (in the order of hundreds) but no instabilities were noticed. This is due to the fact that we use Bézier curves and surfaces that are expressed in terms of Bernstein polynomials, known to be numerically more stable than other choices, as, for instance, monomial or Lagrange bases. Along with this work, we compute derivatives, integrals, additions, and multiplications of Bernstein polynomials, that are stable operations, but we never evaluate polynomials. See Appendix A for further details.

**Table 1:** Comparison of the quadrature-free integration for the 2D and 3D trimmed geometries depicted in Figs. 6 and 7, respectively

	Reference	Quad-free	Relative diff.
2D geo: $M$	2.100230243261870	2.100230243261870	$<10^{-15}$
${\varvec{C}}_M{}\cdot {\varvec{e}}_1$	0.914136125211735	0.914136125211735	$<10^{-15}$
${\varvec{C}}_M{}\cdot {\varvec{e}}_2$	0.859802811586580	0.859802811586580	$<10^{-15}$
3D geo: $M$	0.444790448933688	0.444790378608127	$1.58\times {}10^{-7}$
${\varvec{C}}_M{}\cdot {\varvec{e}}_1$	0.469169723257000	0.469169674580198	$1.03\times {}10^{-7}$
${\varvec{C}}_M{}\cdot {\varvec{e}}_2$	0.400642146493445	0.400642138814180	$1.91\times {}10^{-8}$
${\varvec{C}}_M{}\cdot {\varvec{e}}_3$	0.457115007608867	0.457114990479802	$3.74\times {}10^{-8}$

The mass and the center of mass are evaluated and compared to reference values obtained with an alternative approach based on reparameterization

## Numerical experiments

In this section, we show the performance of the presented quadrature-free approach by means of numerical experiments. In a first set of examples, in Sect. 5.1, we apply the method to the computation of simple integrals in 2D and 3D domains and compare them with standard methods based on the use of boundary-conforming quadrature schemes. Afterwards, in Sect. 5.2 we apply it to the solution of elliptic PDEs using the immersed isogeometric framework presented in Sect. 2.

### Computation of integrals over B-reps

Figures 6 and 7 present two numerical studies used to validate the presented integration strategy. The two-dimensional case, described in Fig. 6, consists of a quadratic Bézier surface which is trimmed by three holes and a vertical curved slice. The three-dimensional case, described in Fig. 7, involves a trimmed domain defined by the intersection of a cube and a free-form cubic trivariate. We compute the mass $M$ and the center of gravity ${\varvec{C}}_M$ of these two geometries, defined by:

53a $$\begin{aligned} M&= \int _{V} \rho ({{\varvec{x}}}) {{\text {d}}{\varvec{x}}}, \end{aligned}$$

53b $$\begin{aligned} {\varvec{C}}_M&= \frac{1}{M} \int _{V} {{\varvec{x}}}\rho ({{\varvec{x}}}) {{\text {d}}{\varvec{x}}}\,, \end{aligned}$$

where the density is considered to be constant $\rho =1$.

Reference values of (53) are obtained through boundary-conformal quadrature schemes created by reparameterizing the interior of *V* with a technique similar to the one presented in [14]. This approach subdivides the domain of integration and leads to integration sub-cells. Standard quadrature rules can then be used to integrate numerically. For the sake of comparison, an overkill number of quadrature points ($64\times 64\times 64$) were used within each integration cell for both examples.

The obtained results are presented in Table 1. For the 2D-geometry (Fig. 6), the computed relative differences, compared with the reparameterization approach, are below $10^{-15}$, i.e., close to machine precision. Nevertheless, for the 3D-geometry (Fig. 7), relative differences of the order of $10^{-7}$ were noticed.

#### Remark 7

We associate the larger differences in the 3D case to the intrinsic tolerances involved in some geometric operations. In this work we employ algorithms provided by Open CASCADE Technology [77] which is an open source C++ library designed for geometric modeling applications. For instance, in the specific case of surface-surface intersections between B-spline or Bézier surfaces, Open CASCADE limits the lowest tolerance to $10^{-7}$, which truncates the achievable accuracy and agrees with the results reported in Table 1. Similar tolerances apply to other non-linear operations. These limitations are not exclusive of Open CASCADE, as similar issues can be found in other commercial and non-commercial geometric kernels available: Tolerances of the order of $10^{-7}$ are more than enough for most of the applications these tools are designed for. On the other hand, we use Irit [78], an open-source geometric modeler, for other 2D operations, as it is the case of the computation of intersections between planar spline curves. The involved tolerances in Irit can be tuned according to our needs, which allows us to reach a higher accuracy for the 2D problem. In addition, it is important to remark that these limitations pollute the geometrical approximation not just for the presented quadrature-free method, but as well for other approaches, as for instance, for surface and volumetric untrimming, as previously discussed in [67]. Nevertheless, we believe that the obtained results confirm the viability of the quadrature-free integration strategy for 3D geometries.

#### Remark 8

For computing the quantities (53) in the case of the 2D-geometry (Fig. 6), the integration procedure can be directly started from Eq. (36), by replacing $\hat{r}_i({\hat{{{\varvec{x}}}}})$ with $\big (\rho \circ {{\varvec{S}}_i}\big )({\hat{{{\varvec{x}}}}})$ and $\big (\rho \circ {{\varvec{S}}_i}\big )({\hat{{{\varvec{x}}}}})\,{{\varvec{S}}_i}({\hat{{{\varvec{x}}}}})\cdot {\varvec{e}}_k,~k=1,2,3$, respectively.

**Fig. 8:**
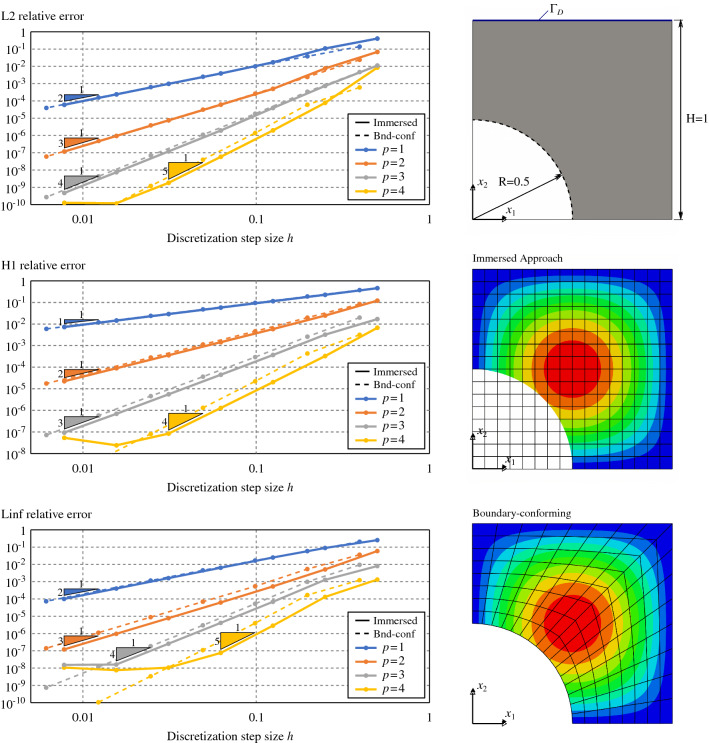
Poisson’s problem over a square with a circular trimmed region

### Immersed isogeometric analysis

In this section, we demonstrate the effectiveness of the quadrature-free approach for solving PDEs in the context of the immersed isogeometric framework presented in Sect. 2. In particular, we perform a series convergence analyses for Poisson’s problem in different 2D (Sect. 5.2.1) and 3D (Sect. 5.2.2) immersed domains. Optimal error convergence rates are retrieved in all the cases. Finally, in Sect. 5.2.3, the flexibility and robustness of the proposed approach is demonstrated in the case of geometries that present a level complexity analogous to the ones found in real industrial applications.

For all the studied cases, we consider the approximated Poisson’s problem (11), previously discussed in Sect. 2. We adopt manufactured solutions:

54 $$\begin{aligned} \begin{aligned} u_{\text {ex}}(x,y)&= \sin (\pi x)\sin (\pi y)&~~\text {in 2D},\\ u_{\text {ex}}(x,y,z)&= \sin (\pi x)\sin (\pi y)\sin (\pi z)&~~\text {in 3D}, \end{aligned} \end{aligned}$$

except for the complex geometries in Sect. 5.2.3. Accordingly, the source and Neumann terms, *f* and *g*, are defined as:

55a $$\begin{aligned} f&= -\Delta {u_{\text {ex}}}\,, \end{aligned}$$

55b $$\begin{aligned} g&= \nabla {u_{\text {ex}}}\cdot {{\varvec{n}}}\,. \end{aligned}$$

The Dirichlet boundary $\Gamma _D$ will be defined for each particular case, and, consequently, Neumann boundary conditions will be applied on $\Gamma _N=\partial \Omega \setminus \Gamma _D$.

The choice of such regular functions as target solutions (Eq. 54) is motivated by the aim of focusing our study on the consistency error, mainly controlled by numerical integration and geometric representation errors, while keeping the discretization error small. The approximation properties of trimmed spline spaces for the solution of elliptic PDEs have been previously studied in [67].

**Fig. 9:**
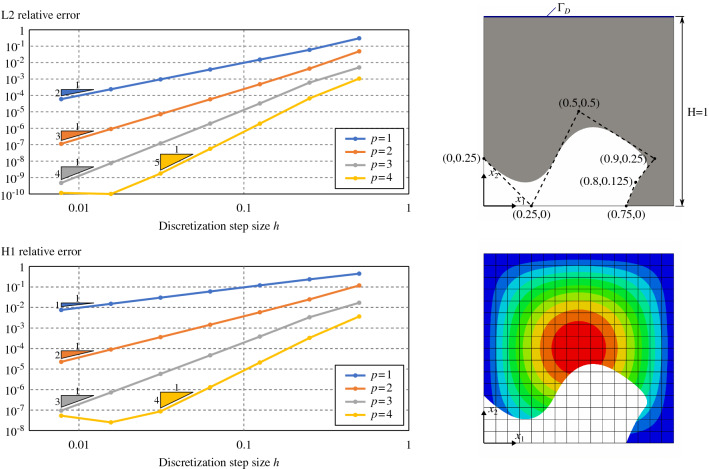
Poisson’s problem over a square with a free-form trimmed region

#### Poisson’s problem for 2D trimmed-geometries

**Fig. 10:**
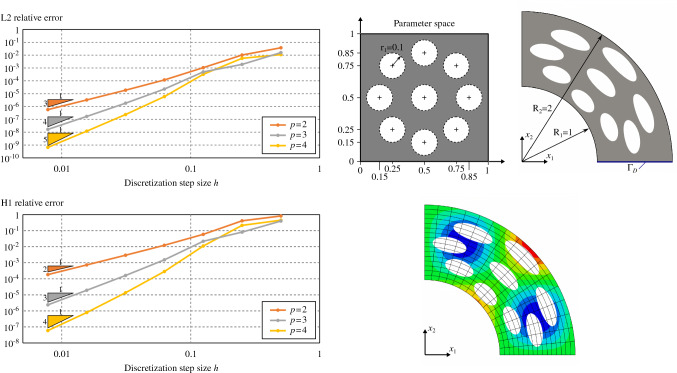
Poisson’s problem over a one-quarter annulus with several holes

Let us first tackle the Poisson’s problem for several two-dimensional problems:

- a square with a circular hole (Fig. 8),
- a square with a free-form hole (Fig. 9),
- a multi-perforated quarter annulus (Fig. 10).

Several solution degrees are considered: i.e., from $p=1$ for the trimmed squares, and $p=2$ for the annulus, to $p=4$. Importantly, the presence of conic sections require to perform some geometric approximations such that the integrals in the finite element operators involve only non-rational polynomials. As already discussed in Remark 2, to do so we rely on the results proven in [67] which reveal that approximating the elements’ geometry using degree *p* leads to optimal numerical results. Therefore, Béziers of degree *p* are used to approximate the rational geometrical quantities at the element level.

In addition, it is important to remark the presence of a non-identity mapping in the problem depicted in Fig. 10. This leads to the introduction of an extra non-polynomial term in the bilinear form (see Remark 1) that is approximated through a local polynomial projection, as discussed in Sect. 2.2.

The $H^1$ and $L^2$ relative norms of the solution errors are evaluated along with the analyses. Optimal convergence rates, *p* and $p+1$, respectively, are retrieved for the three cases, see Figs. 8,  9, and 10.

In the case of the plate with a hole case (Fig. 8), the $L^\infty$ norm was also studied observing an optimal convergence behavior^2^.

In addition, for that particular test case, the results of the proposed immersed approach were compared against the ones obtained using a boundary-fitted method. As it can be seen in Fig. 8, for a fixed element size *h*, both results are comparable in terms of accuracy for all the computed norms.

In Figs.  8,  9, and 10, the $H^1$ and $L^2$ norms were computed using tensor-product Gauss-Legendre quadrature rules with $p+6$ points per direction for the active non-cut elements, including the elements of the boundary-fitted method. For the cut-elements, the norms were evaluated by means of the reparameterization approach already employed during the validation of the integrals computed in Sect. 5.1, using $p+6$ points per direction for every integration sub-cell. On the other hand, in Fig. 8, the $L^\infty$ norm was computed using 64 equally distributed points along each direction for every non-cut element and integration sub-cell.

The numerical solutions obtained with the quadrature-free approach enable to validate the present methodology for two-dimensional cases.

Nevertheless, it is important to remark that for the finest discretizations in the case $p=4$, the error reaches a *plateau* (around $10^{-10}$ for the relative $L^2$ error norms, and around $10^{-8}$ for the relative $H^1$ and $L^\infty$ norms). For those cases, the discretization error becomes lower than the error induced by geometrical operations as, for instance, the slicing of the domain $\Omega$ into elements. See the related discussion in Remark 7. Similar *plateaux* were observed in [67, 79].

#### Poisson’s problem for simple 3D trimmed-geometries

**Fig. 11:**
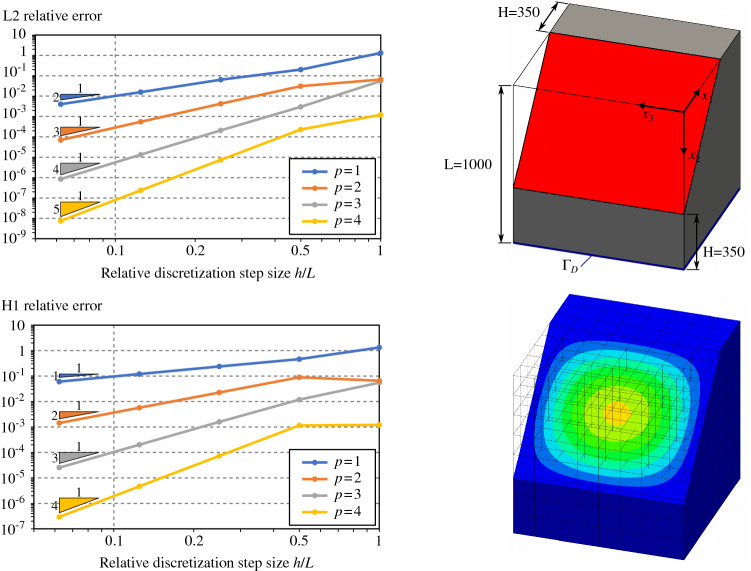
Poisson’s problem over a cube with a planar trimmed region

**Fig. 12:**
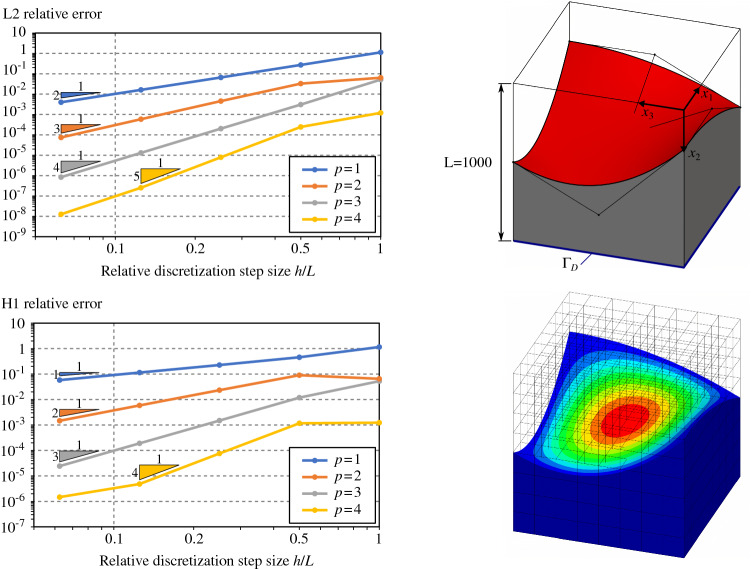
Poisson’s problem over a cube with a free-form trimmed region

**Fig. 13:**
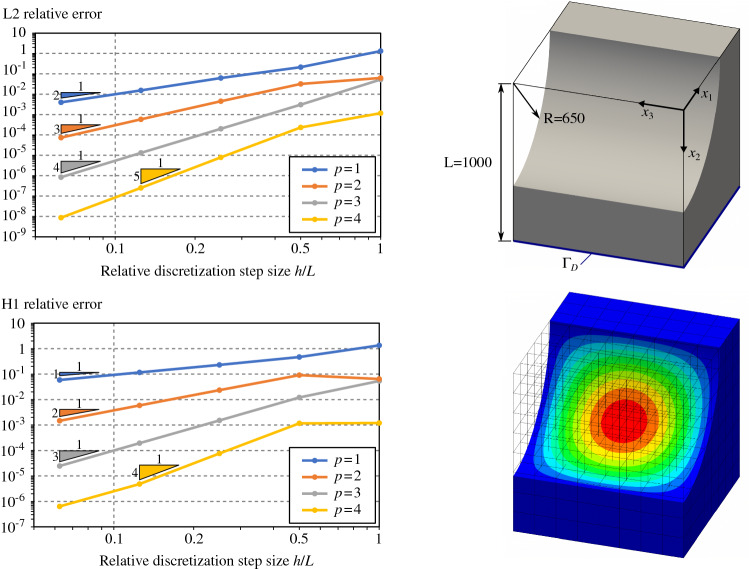
Poisson’s problem over a cube with a cylindrical trimmed region

**Fig. 14:**
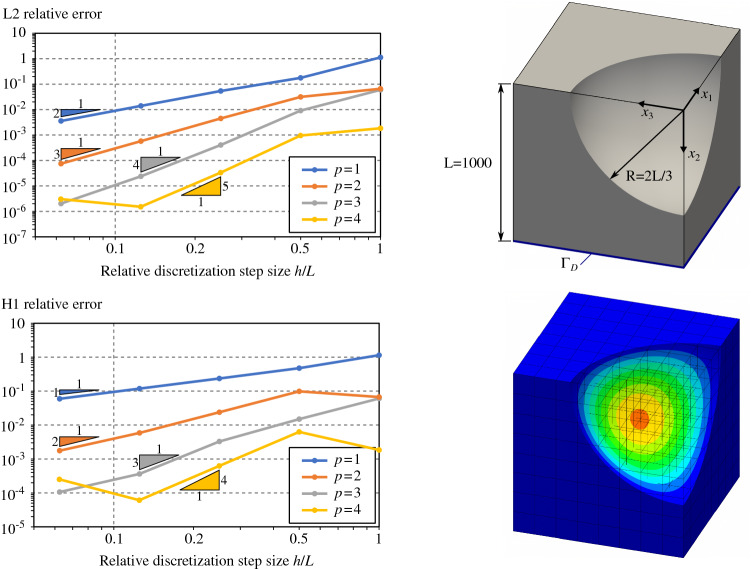
Poisson’s problem over a cube with a spherical trimmed region

To go one step further, we perform several analyses on three-dimensional trimmed domains. We consider four trimmed domains with several levels of complexity. Each of them consists in a cube with length $L=1000$ with different trimmed regions:

- a simple planar cut (Fig. 11),
- a free-form cut (Fig. 12) which is defined by a bi-quadratic surface with the following control points:
  $$\begin{aligned} \mathbf {P}= \begin{pmatrix} 0 &{} L/2 &{} L &{} 0 &{} L/2 &{} L &{} 0 &{} L/2 &{} L \\ L/4 &{} L/4 &{} L/2 &{} 3L/4 &{} L/2 &{} L/4 &{} L/2 &{} 3L/4 &{} L/4 \\ 0 &{} 0 &{} 0 &{} L/2 &{} L/2 &{} L/2 &{} L/2 &{} 3L/4 &{} L/4 \end{pmatrix} \end{aligned}$$
- one-quarter of a cylinder (Fig. 13),
- one-eighth of a sphere (Fig. 14).

As for the 2D-cases, we study the convergence rate in both $H^1$ and $L^2$ relative norms for several spline degrees. The norms are again evaluated via a reparameterization procedure. The obtained results confirm the theoretical expectations: Optimal convergence rates are confirmed.

The curves created by Open CASCADE [77] during the surface-surface intersections are represented as B-splines of high degree and possibly rational. Such high order curves may lead to very high degrees during the polynomial compositions, as detailed in Sect. 4.3. As for the 2D-cases, and according to Remark 2, it is always possible to approximate at the element level those geometrical entities with Béziers of degree equal to the solution degree. This turns out to be mandatory in the case of rational curves and surfaces. For all trimmed cubes included in this section, the curves arising from surface-surface intersections were approximated at element level using Bézier curves of degree *p*. In the same way, for the cases in Figs. 13 and 14, the underlying rational surfaces were also approximated at element level with Bézier surfaces of degree *p* along both parametric directions.

Thus, for the case of the planar cut described in Fig. 11, the accuracy of the surface-surface intersections is very good due to the simplicity of the underlying geometric slices (just straight lines). Consequently, in this particular example, the convergence rates are optimal, even for $p=4$ over the finest mesh (see again Fig. 11). The cube with the cylindrical removal also presents optimal convergence rates as shown in Fig. 13. We observe that for this geometry, the surface–surface intersections are also precisely computed using Open CASCADE [77]: The intersection curves are straight lines in the parametric domain of the cylindrical surface. However, in the case of the free-form trimmed cube (Fig. 12) and spherical trimmed cube (Fig. 14), the optimal convergence rates start to deteriorate for the finest discretization and $p=4$. This is due to the fact that the intersection curves are no longer straight lines in the parametric domain of the trimming surfaces, thus, they are strongly influenced by the used tolerance values, as already discussed in Remark 7. Let us mention that similar results were previously observed in [67].

Let us also study the involved polynomial degrees for the four examples included in this section according to the estimation detailed in Sect. 4.3. Applying the quadrature-free approach to solve the Poisson’s problem (11), we can identify the polynomial integrand *a* [recall Eq. (26)] with the term $B^{\mathbf {0}}_k \big (\nabla {N_i}\otimes \nabla {N_j}\big )|_{Q}\in \mathbb {Q}_{2p,\,2p,\,2p}$ (Eq. (18), where we assumed $\bar{{\varvec{K}}}$ to be the identity and therefore the projection degrees to be $\mathbf {q}=(0,0,0)$). Considering, as discussed above, that the degrees of approximated surfaces and curves are $s=c=p$, the final degree of the polynomial term $\tilde{t}_{i,j}$ becomes [recall Eq. (52)]:

56 $$\begin{aligned} \tilde{t}_{i,j} \in \mathbb {Q}_{w},\text {with }w=12 p^3 + 6 p^2 - 1. \end{aligned}$$

Unsurprisingly, the degree *w* is very high: $w=\left\{ 17,\,119,\,377,\,863\right\}$ for $p=\left\{ 1,\,2,\,3,\,4\right\}$, respectively. Nevertheless, despite these high orders, no instabilities were noticed in the results of Figs. 11, 12, 13 and 14. As previously discussed in Sect. 4.3, this is due to the fact that the proposed integration strategy does not require polynomial evaluations. An in-depth discussion can be found in Appendix A. Nevertheless, we noticed that the results start to deteriorate for degree $p=5$, for which the total polynomial degree of $\tilde{t}_{i,j}$ becomes $w=1649$. This is due to the fact that the values of the binomial coefficients that appear in the Bernstein polynomial multiplications and compositions (Eqs. 66,67,71,76) are very large. Double precision variables (64bits) are not enough for representing those numbers with sufficient precision, which pollutes all subsequent computations.

Finally, regarding the computational cost, we observe that the slicing process as well as the posterior approximation step are not particularly expensive operations. Thus, for instance, for the example of Fig. 14 with $16\times 16\times 16$ elements, the intersection of the geometry with the Cartesian background grid took around 10.8 seconds running in a single core of an Intel i7-8559U 2.7 GHz processor. For that specific case, the approximation stage, took 3.3, 3.9, 4.6, and 5.8 seconds, for degrees $p=\left\{ 1,\,2,\,3,\,4\right\}$, respectively. It is important to note that these operations (slicing and approximation) are easily parallelizable, and therefore, the total time can be significantly reduced by using all the cores available on modern processor architectures.

#### Poisson’s problem on complex 3D trimmed-geometries

**Fig. 15:**
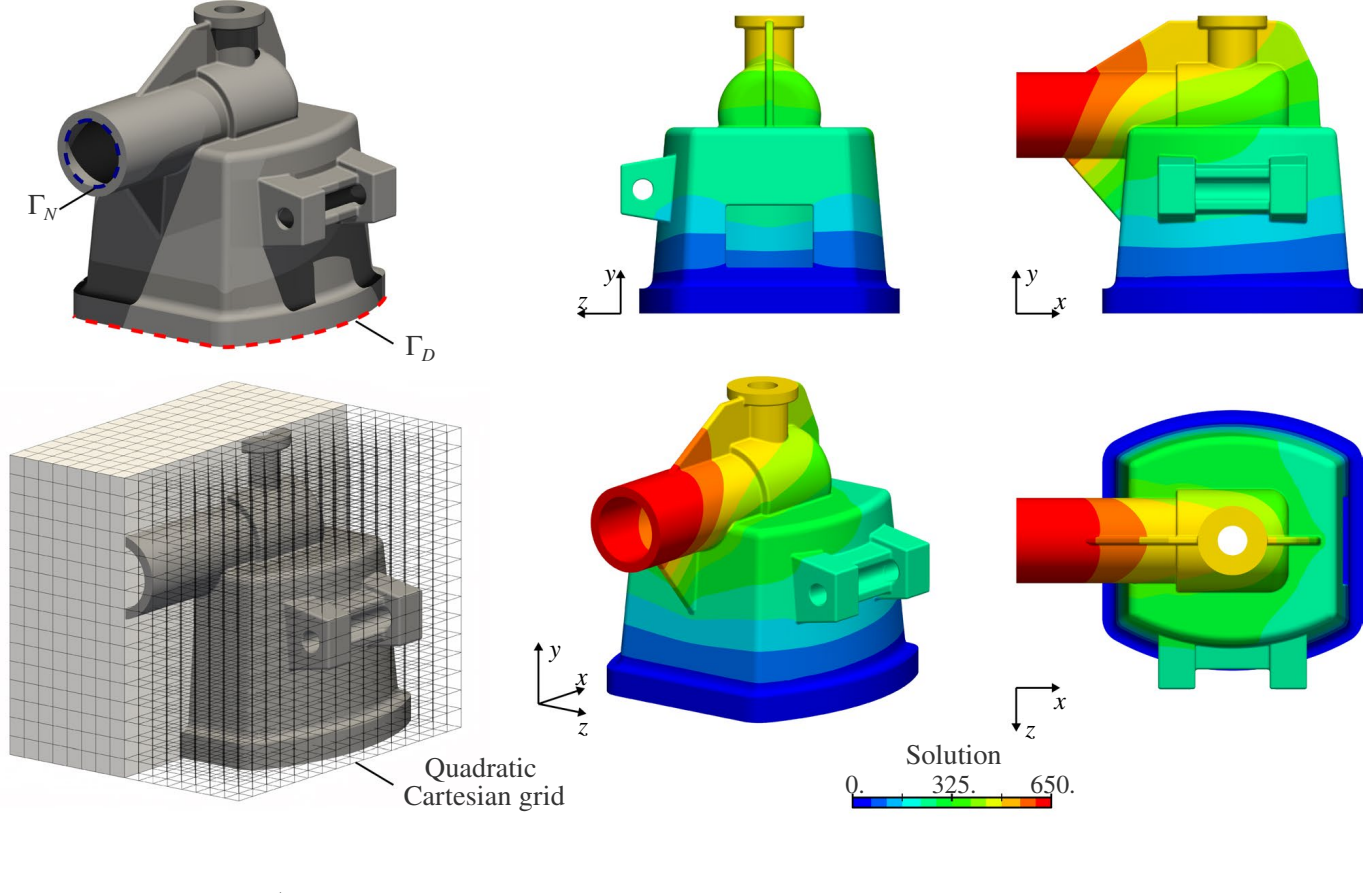
Poisson’s problem over the first complex CAD geometry

**Fig. 16:**
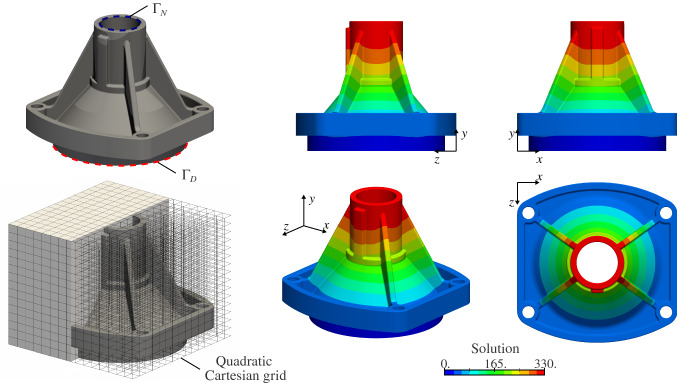
Poisson’s problem over the second complex CAD geometry

To show the viability of the quadrature-free approach to handle complex 3D geometries, we consider the two CAD models shown in Figs. 15 and 16. These B-Rep geometries have been extracted from the Open CASCADE database [77]. Generating a boundary-conforming volumetric parameterization of these geometries is far from a simple task. Instead, the B-Rep models are immersed into Cartesian grids (see Sect. 2). The solutions are discretized with $C^1$-continuous quadratic B-spline basis functions. Again, we solve Poisson’s problem with homogeneous Dirichlet boundary condition applied on the bottom surfaces and a constant Neumann boundary condition inside the cylindrical tubes (see again Figs. 15 and 16). In order to build the finite element operators, the presented quadrature-free approach is applied. The obtained solutions are depicted in Figs. 15 and 16.

The stiffness matrices associated with these examples were ill-conditioned due to the presence of small cut elements. In order to solve this issue, the associated linear systems were preconditioned using a Jacobi preconditioner as described in [61].

Regarding the computational cost of the geometric operations, for the example in Fig. 15 the slicing process and subsequent approximation stage took 55.0 and 84.8 seconds, respectively; while 46.2 and 68.5 seconds were measured, respectively, for the test in Fig. 16. As in the previous sections, these times were obtained using a single core of an Intel i7-8559U 2.7 GHz processor.

We believe that these two complex geometries highlight the viability of the developed approach to deal with designs of industrial complexity level.

## Conclusions

We have presented a novel approach for the solution of partial differential equations on B-Rep geometries by means of immersed isogeometric discretizations that do not require quadrature schemes. For such purpose, we developed a new quadrature-free technique for the evaluation of integrals with polynomial integrands over B-Reps enclosed by trimmed non-rational spline surfaces.

This technique is based on two successive applications of the divergence theorem, transforming 3D integrals into line integrals that are eventually computed analytically. The involved steps require the creation and manipulation of (potentially) very high-degree polynomials. Nevertheless, we do not perform explicit evaluation of such functions, but just operations as additions or multiplications (using Bernstein bases), that are known to be more stable. The accuracy of this integration method has been verified numerically by evaluating integrals of low order polynomials over 2D and 3D domains and comparing the obtained results against reference solutions computed through boundary-conformal quadrature schemes.

To apply such an integration method to the resolution of PDEs over CAD models using immersed Galerkin discretizations, we transform the integrands of the finite element operators into polynomials. Thus, relying on [66] we create local polynomial approximations of those integrands for every element. In addition, according to [67], we also approximate at element level the rational B-splines, that may define the geometry, as non-rational Bézier curves and surfaces. This opens the door to the application of the method to B-Reps enclosed by rational splines.

The combination of the results in [66, 67] theoretically guarantees the optimal approximation properties of the proposed method for elliptic problems. This approach is directly extendable to other non-elliptic problems, however, suitable approximation properties are not backed up by theoretical evidences.

A series of numerical experiments support our claims. Thus, the method’s performance is illustrated by a series of elliptic problems on immersed 2D and 3D geometries, some of which present rational geometries. Optimal convergence rates were confirmed in all the cases. Finally, and to prove the potential of the method, its real applicability is demonstrated with a couple of 3D B-Rep models with an industrial level of geometrical complexity.

In this work, we particularize our study to the case of isogeometric discretizations. Nevertheless, the ideas behind are straightforwardly extendable to other immersed methods as, for instance, the finite cell method or CutFEM/IGA [31–33], or to other discretization techniques like XFEM or high-order virtual element [80, 81] methods. In addition, the quadrature-free integration could be also handy for the evaluation of the right-hand-side integrals involved in the moment fitting techniques [53].

## Appendix: A Bernstein polynomials

In this Appendix we discuss the construction of polynomials using Bernstein bases. We first introduce, in A.1, the Bernstein basis, its main properties, and the construction of univariate polynomials. Afterwards, in A.2, we discuss its generalization to the case of tensor-product polynomials. And finally, in A.3 we present the case of multi-dimensional vector polynomials. Most of the constructions detailed in this Appendix are rather classical and can be found, for instance, in [82].

### A.1 Bernstein basis and univariate polynomials

Let us first introduce the Bernstein polynomials basis for a degree $p\ge 0$:

57 $$\begin{aligned} B_{i}^p(t) = \left( {\begin{array}{c}p\\ i\end{array}}\right) t^i(1-t)^{p-i}, ~~i=0,\dots ,p. \end{aligned}$$

It is well-known that this basis constitutes an appealing alternative to monomials in terms of numerical stability when it comes to floating-point operations [82].

In addition, the Bernstein basis presents some handy properties that simplify the manipulation of polynomials. For instance, their derivatives can be easily computed as a function of lower degree polynomials. Thus, for $p>0$:

58a $$\begin{aligned} \frac{{\text {d}}B_{0}^p(t)}{{\text {d}}t}&= - p B_{0}^{p-1}(t)\,, \end{aligned}$$

58b $$\begin{aligned} \frac{{\text {d}}B_{i}^p(t)}{{\text {d}}t}&= p B_{i-1}^{p-1}(t) - p B_{i}^{p-1}(t)\quad \text {for }0<i<p\,, \end{aligned}$$

58c $$\begin{aligned} \frac{{\text {d}}B_{p}^p(t)}{{\text {d}}t}&= p B_{p-1}^{p-1}(t)\,. \end{aligned}$$

In the same way, their primitives can be computed using polynomials of higher degree:

59 $$\begin{aligned} \int B_{i}^p(t){\text {d}}t = \frac{1}{p+1}\sum _{j=i+1}^{p+1} B_{j}^{p+1}(t), \end{aligned}$$

that yields:

60 $$\begin{aligned} \int _{0}^{1} B_{i}^p(t){\text {d}}t = \frac{1}{p+1}. \end{aligned}$$

In addition, any Bernstein polynomial of degree $p-1$, with $p>0$, can be expressed as a combination of polynomials of degree *p* as:

61 $$\begin{aligned} B_{i}^{p-1}(t) = \frac{p-i}{p} B_{i}^{p}(t) + \frac{i+1}{p} B_{i+1}^{p}(t). \end{aligned}$$

Using the Bernstein basis, a univariate real polynomial *f*(*t*) of degree *p* can be expressed as:

62 $$\begin{aligned} f(t) = \sum _{i=0}^p B_{i}^p(t) f_i, \end{aligned}$$

where $f_i\in \mathbb {R}$. Applying (58), (59), (60), and (61) to each Bernstein basis function of the polynomial *f*(*t*), it is straightforward to compute the derivative of *f*(*t*), its antiderivative, integrate it over the domain [0, 1], and express it using a basis of degree $p+1$, respectively. In particular, due to its particular interest in this work, the integral of *f*(*t*) over the domain [0, 1] is detailed:

63 $$\begin{aligned} \int _{0}^{1} f(t){\text {d}}t = \frac{1}{p+1}\sum _{i=0}^{p}f_i. \end{aligned}$$

This result can be directly applied to the computation of the integral (45), in Sect. 4. We remark that in this operation no polynomial evaluations are involved, simply the linear combination of the coefficients $f_i$, what makes this computation stable even for high degree polynomials.

Let us know introduce now a second polynomial *g*(*t*) of degree $q\ge 0$:

64 $$\begin{aligned} g(t) = \sum _{i=0}^q B_{i}^q(t) g_i. \end{aligned}$$

In the case $q=p$, the addition (subtraction) of *f*(*t*) and *g*(*t*) it is easily computed by adding (subtracting) their coefficients:

65 $$\begin{aligned} f(t) \pm g(t) = \sum _{i=0}^p B_{i}^q(t) (f_i \pm g_i). \end{aligned}$$

On the other hand, if $q < p$, *g*(*t*) must be firstly written in the Bernstein basis of degree *p*, applying (61) $p-q$ times, and then the expression (65) can be directly used.

The multiplication of polynomials is another operation that is extensively used in Sect. 4. The product *f*(*t*)*g*(*t*) yields a new polynomial of degree $p+q$ that can be computed as:

66 $$\begin{aligned} f(t)\,g(t) = \sum _{i=0}^{p+q}B_{i}^{p+q}(t)\left (\sum _{j=\max (0,i-q)}^{\min (p,i)} \frac{\left( {\begin{array}{c}p\\ j\end{array}}\right) \left( {\begin{array}{c}q\\ i-j\end{array}}\right) }{\left( {\begin{array}{c}p+q\\ i\end{array}}\right) } f_j g_{i-j}\right). \end{aligned}$$

Based on that, the composition of two polynomials $f\circ g(t)$ is expressed as:

67 $$\begin{aligned} \begin{aligned} f\circ g(t)&= \sum _{i=0}^{p} B_{i}^{p}\left( g(t)\right) f_i\\&= \sum _{i=0}^{p} \left( {\begin{array}{c}p\\ i\end{array}}\right) g(t)^i \left( 1-g(t)\right) ^{p-i}f_i, \end{aligned} \end{aligned}$$

where the terms $g(t)^i \left( 1-g(t)\right) ^{p-i}$, $i=1,\dots ,p$, can be evaluated by means of the polynomials product expression (66).

### A.2 Multivariate polynomials

The univariate construction (62) can be extended to the case of *m*-dimensional tensor-product polynomials as:

68 $$\begin{aligned} h(t_1,t_2,\dots ,t_m) \nonumber \\ =\sum _{i_1=0}^{p_1}\sum _{i_2=0}^{p_2}\cdots \sum _{i_m=0}^{p_m} B_{i_1}^{p_1}(t_1) B_{i_2}^{p_2}(t_2) \cdots B_{i_m}^{p_m}(t_m) h_{{\varvec{i}}}, \end{aligned}$$

where $(p_1,p_2,\ldots ,p_m)$ are the non-negative degrees along the *m* parametric directions, and ${\varvec{i}}=(i_1,i_2,\ldots ,i_m)$ the multi-index accounting for all the univariate indices. Operations defined for univariate polynomials, as derivatives (58), primitives (59), or degree raising (61), can now be applied for every parametric direction independently. For instance, the computation of antiderivatives along different directions is required in Eqs. (30) and (39). On the other hand, the integral of $h(t_1,\dots ,t_m)$ over a domain $[0,1]^m$ can be easily computed as:

69 $$\begin{aligned} \int _{0}^{1}\int _{0}^{1}\dots \int _{0}^{1} h(t_1, t_2,\dots ,t_m){\text {d}}t_1{\text {d}}t_2\dots {\text {d}}t_m \nonumber \\= \frac{1}{\prod ^{m}_{j=1} \left( p_j+1\right) } \sum _{{\varvec{i}}}h_{{\varvec{i}}}. \end{aligned}$$

As discussed in Remark 4, for the case of non-trimmed Bézier patches, the surface integral (36) can be directly computed using the expression above. The same applies to the integral (26) in the case the integration domain is a unit cube (what is applicable to integration over non-cut elements $Q\in {\mathcal {T}\,}^{\text {int}}_h(\Omega )$ as discussed in Sect. 2). This is the case of the evaluation of integrals over the non-cut elements discussed in Sect. 2. We also remark here that, as for the univariate case (60), no polynomial evaluations are required for computing this integral, only a linear combination of the coefficients $h_{{\varvec{i}}}$.

We now consider a second *m*-dimensional polynomial $l(t_1,\dots ,t_m)$ with non-negative degrees $(q_1,\dots ,q_m)$:

70 $$\begin{aligned} &l(t_1,t_2,\dots ,t_m)\nonumber \\ & \quad =\sum _{i_1=0}^{q_1}\sum _{i_2=0}^{q_2}\cdots \sum _{i_m=0}^{q_m} B_{i_1}^{q_1}(t_1) B_{i_2}^{q_2}(t_2) \cdots B_{i_m}^{q_m}(t_m) l_{{\varvec{i}}}, \end{aligned}$$

where $l_{{\varvec{i}}}\in \mathbb {R}$. The multiplication of two *m*-dimensional polynomials, analogously to (66), results in a polynomial with degrees $(p_1+q_1,\dots ,p_m+q_m)$ that can be computed as:

71 $$\begin{aligned}&h(t_,\dots ,t_m)\,l(t_,\dots ,t_m) = \sum _{i_1=0}^{p_1+q_1} \cdots \sum _{i_m=0}^{p_m+q_m} B_{i_1}^{p_1+q_1}(t_1)\cdots B_{i_m}^{p_m+q_m}(t_m)\nonumber \\&\quad \sum _{j_1=\max (0,i_1-q_1)}^{\min (p_1,i_1)} \cdots \sum _{j_m=\max (0,i_m-q_m)}^{\min (p_m,i_m)} \frac{\left( {\begin{array}{c}p_1\\ j_1\end{array}}\right) \left( {\begin{array}{c}q_1\\ i_1-j_1\end{array}}\right) }{\left( {\begin{array}{c}p_1+q_1\\ i_1\end{array}}\right) } \cdots \nonumber \\&\quad \frac{\left( {\begin{array}{c}p_m\\ j_m\end{array}}\right) \left( {\begin{array}{c}q_m\\ i_m-j_m\end{array}}\right) }{\left( {\begin{array}{c}p_m+q_m\\ i_m\end{array}}\right) } h_{j_1,\dots ,j_m} l_{i_1-j_1,\dots ,i_m-j_m}. \end{aligned}$$

### A.3 Vector polynomials

The univariate and multivariate polynomials studied above constitute the foundation for the construction of Bézier curves, surfaces, and other higher dimensional geometric objects. In particular, following the polynomial constructions (62) and (68), Bézier curves and surfaces can be expressed as:

72a $$\begin{aligned} {\varvec{c}}(t)&= \sum _{i=0}^p B_{i}^p(t) {\varvec{c}}_i, \end{aligned}$$

72b $$\begin{aligned} {\varvec{S}}(t_1, t_2)&= \sum _{i_1=0}^{p_1}\sum _{i_2=0}^{p_2} B_{i_1}^{p_1}(t_1)B_{i_2}^{p_2}(t_2) {\varvec{S}}_{i_1,i_2}, \end{aligned}$$

where ${\varvec{c}}_i,{\varvec{S}}_{i_1,i_2}\in \mathbb {R}^d$ and *d* is the spatial dimension. The single coordinate components of ${\varvec{c}}$ and ${\varvec{S}}$ are themselves scalar polynomials and can expressed as:

73a $$\begin{aligned} c_k(t)&= {\varvec{c}}(t)\cdot {\varvec{e}}_k = \sum _{i=0}^p B_{i}^p(t) {\varvec{c}}_i\cdot {\varvec{e}}_k, \end{aligned}$$

73b $$\begin{aligned} S_k(t_1,t_2)&= {\varvec{S}}(t_1,t_2)\cdot {\varvec{e}}_k\nonumber \\&= \sum _{i_1=0}^{p_1} \sum _{i_2=0}^{p_2} B_{i_1}^{p_1}(t_1) B_{i_2}^{p_2}(t_2) {\varvec{S}}_{i_1,i_2}\cdot {\varvec{e}}_k, \end{aligned}$$

for $k=1,\dots ,d$, and where ${\varvec{e}}_k$ are the unit vectors along the Cartesian directions.

Thus, operations like partial derivatives, or cross and scalar products between Bézier curves and surfaces, like the ones used in Sect. 4, can be carried out by using its individual coordinate components (73) and combining them according to the operations detailed in previous sections for scalar univariate and multivariate polynomials. Among all the operations, due to its higher complexity, in what remains we detail the composition between multivariate Béziers.

We consider two multivariate Béziers ${\varvec{F}}:\mathbb {R}^s\rightarrow \mathbb {R}^d$ and ${\varvec{G}}:\mathbb {R}^m\rightarrow \mathbb {R}^s$ of the form:

74a $$\begin{aligned} {\varvec{F}}(r_1,\dots ,r_s)&= \sum _{i_1=0}^{p_1}\cdots \sum _{i_s=0}^{p_s} B_{i_1}^{p_1}(r_1) \cdots B_{i_s}^{p_s}(r_s) {\varvec{F}}_{{\varvec{i}}}\,, \end{aligned}$$

74b $$\begin{aligned} {\varvec{G}}(t_1,\dots ,t_m)&= \sum _{j_1=0}^{q_1}\cdots \sum _{j_m=0}^{q_m} B_{j_1}^{q_1}(t_1) \cdots B_{j_m}^{q_m}(t_m) {\varvec{G}}_{{\varvec{j}}}\,, \end{aligned}$$

that have non-negative degrees $(p_1,p_2,\dots ,p_s)$ and $(q_1,q_2,\dots ,q_m)$, respectively. ${\varvec{G}}_{{\varvec{i}}}\in \mathbb {R}^s$ and ${\varvec{F}}_{{\varvec{i}}}\in \mathbb {R}^d$ are the associated control points, and $\mathbf {i}=(i_1,i_2,\dots ,i_s)$ and $\mathbf {j}=(j_1,j_2,\dots ,j_m)$ the corresponding multi-indices. We want to compute the composition ${\varvec{F}}\circ {\varvec{G}} (t_1,\dots ,t_m):\mathbb {R}^m\rightarrow \mathbb {R}^d$. Working with the coordinate components $G_k(t_1,\dots ,t_m) = {\varvec{G}}(t_1,\dots ,t_m)\cdot {\varvec{e}}_k,~k=1,\dots ,s$, we obtain:

75 $$\begin{aligned} {\varvec{F}}\circ {\varvec{G}}(t_1,\dots ,t_m) = \sum _{i_1=0}^{p_1}\cdots \sum _{i_s=0}^{p_s} B_{i_1}^{p_1}\circ G_1(t_1,\dots ,t_m) \nonumber \\\cdots B_{i_s}^{p_s}\circ G_s(t_1,\dots ,t_m) {\varvec{F}}_{{\varvec{i}}}. \end{aligned}$$

Every term $B_{i_k}^{q_k}\circ G_k(t_1,\dots ,t_m),~k=1,\dots ,s$, is the composition between a univariate Bernstein polynomial and a *m*-dimensional scalar polynomial expressed in a tensor-product Bernstein basis:

76 $$\begin{aligned} & B_{i_k}^{p_k}\circ G_k(t_1,\dots ,t_m) \nonumber \\ & \quad = \left( {\begin{array}{c}p_k\\ i_k\end{array}}\right) G_k\left( t_1,\dots ,t_m\right) ^{i_k}\left( 1-G_k\left( t_1,\dots ,t_m\right) \right) ^{p_k-i_k}, \end{aligned}$$

where the products are computed performing multiplications between multi-dimensional scalar polynomials, detailed in Eq. (71).

## Footnotes

1 Due to the fact that $\mathbb {Q}_{p +q,\,p +q,\,p +q}\subset \mathbb {Q}_{2 p +q,\,2 p +q,\,2 p +q}$, the integrals $\int _{Q\cap \Omega } B^{\mathbf {s}}_{\beta } {\text {d}}Q$ in (19b) can be computed as linear combinations of the integrals $\mathsf {I}^{3D }_{Q,\alpha }.$

2 The $L^\infty$ norm of a quantity $f\in L^\infty (\Omega )$ is known to be lower bounded by the $L^2$ norm as $||f ||^2_{L^2(\Omega )} \le C ||f ||^2_{L^\infty (\Omega )}$, where *C* is a constant equal to the volume of the domain $\Omega$.

## References

[1] Hughes TJR, Cottrell JA, Bazilevs Y (2005). Isogeometric analysis: CAD, finite elements, NURBS, exact geometry and mesh refinement. Comput Methods Appl Mech Eng.

[2] Cottrell JA, Hughes TJR, Bazilevs Y (2009). Isogeometric analysis: toward integration of CAD and FEA..

[3] Liu G (2009) Meshfree methods. CRC Press. 10.1201/9781420082104

[4] Bazilevs Y, da Veiga LB, Cottrell JA, Hughes TJR, Sangalli G (2006). Isogeometric analysis: approximation, stability and error estimates for h-refined meshes. Math Models Methods Appl Sci.

[5] Buffa A, Rivas J, Sangalli G, Vázquez R (2011). Isogeometric discrete differential forms in three dimensions. SIAM J Numer Anal.

[6] Hiemstra R, Toshniwal D, Huijsmans R, Gerritsma M (2014). High order geometric methods with exact conservation properties. J Comput Phys.

[7] Lipton S, Evans J, Bazilevs Y, Elguedj T, Hughes TJR (2010). Robustness of isogeometric structural discretizations under severe mesh distortion. Comput Methods Appl Mech Eng.

[8] Herrema AJ, Wiese NM, Darling CN, Ganapathysubramanian B, Krishnamurthy A, Hsu M-C (2017). A framework for parametric design optimization using isogeometric analysis. Comput Methods Appl Mech Eng.

[9] Antolin P, Buffa A, Cohen E, Dannenhoffer JF, Elber G, Elgeti S, Haimes R, Riesenfeld R (2019). Optimizing micro-tiles in micro-structures as a design paradigm. Comput Aided Des.

[10] Hafner C, Schumacher C, Knoop E, Auzinger T, Bickel B, Bächer M (2019). X-CAD: optimizing CAD models with extended finite elements. ACM Trans Graph.

[11] Hirschler T, Bouclier R, Duval A, Elguedj T, Morlier J (2020). A new lighting on analytical discrete sensitivities in the context of IsoGeometric shape optimization. Arch Comput Methods Eng.

[12] Akhras HA, Elguedj T, Gravouil A, Rochette M (2016). Isogeometric analysis-suitable trivariate NURBS models from standard B-Rep models. Comput Methods Appl Mech Eng.

[13] Hinz J, Möller M, Vuik C (2018). Elliptic grid generation techniques in the framework of isogeometric analysis applications. Comput Aided Geom Des.

[14] Massarwi F, Antolin P, Elber G (2019). Volumetric untrimming: Precise decomposition of trimmed trivariates into tensor products. Comput Aided Geom Des.

[15] Maquart T, Wenfeng Y, Elguedj T, Gravouil A, Rochette M (2020). 3D volumetric isotopological meshing for finite element and isogeometric based reduced order modeling. Comput Methods Appl Mech Eng.

[16] Wang W, Zhang Y, Xu G, Hughes TJR (2012). Converting an unstructured quadrilateral/hexahedral mesh to a rational T-spline. Comput Mech.

[17] Wei X, Zhang YJ, Toshniwal D, Speleers H, Li X, Manni C, Evans JA, Hughes TJR (2018). Blended B-spline construction on unstructured quadrilateral and hexahedral meshes with optimal convergence rates in isogeometric analysis. Comput Methods Appl Mech Eng.

[18] Xia S, Qian X (2017). Isogeometric analysis with Bézier tetrahedra. Comput Methods Appl Mech Eng.

[19] Koh K. J, Toshniwal D, Cirak F An optimally convergent smooth blended B-spline construction for unstructured quadrilateral and hexahedral meshes, arXiv preprint arXiv:2111.04401

[20] Peters J (2020). Refinable tri-variate $${C}^1$$ splines for box-complexes including irregular points and irregular edges. Comput Aided Geom Des.

[21] Rank E, Ruess M, Kollmannsberger S, Schillinger D, Düster A (2012). Geometric modeling, isogeometric analysis and the finite cell method. Comput Methods Appl Mech Eng.

[22] Legrain G (2013). A NURBS enhanced extended finite element approach for unfitted CAD analysis. Comput Mech.

[23] Breitenberger M, Apostolatos A, Philipp B, Wüchner R, Bletzinger K-U (2015). Analysis in computer aided design: Nonlinear isogeometric B-Rep analysis of shell structures. Comput Methods Appl Mech Eng.

[24] Hsu M-C, Wang C, Xu F, Herrema AJ, Krishnamurthy A (2016). Direct immersogeometric fluid flow analysis using B-rep CAD models. Comput Aided Geom Des.

[25] Guo Y, Heller J, Hughes TJR, Ruess M, Schillinger D (2018). Variationally consistent isogeometric analysis of trimmed thin shells at finite deformations, based on the step exchange format. Comput Methods Appl Mech Eng.

[26] Wassermann B, Kollmannsberger S, Yin S, Kudela L, Rank E (2019). Integrating CAD and numerical analysis: “dirty geometry” handling using the finite cell method. Comput Methods Appl Mech Eng.

[27] Marussig B, Hughes TJR (2017). A review of trimming in isogeometric analysis: challenges, data exchange and simulation aspects. Arch Comput Methods Eng.

[28] Peskin CS (2002). The immersed boundary method. Acta Numer.

[29] Düster A, Parvizian J, Yang Z, Rank E (2008). The finite cell method for three-dimensional problems of solid mechanics. Comput Methods Appl Mech Eng.

[30] Schillinger D, Dede L, Scott MA, Evans JA, Borden MJ, Rank E, Hughes TJR (2012). An isogeometric design-through-analysis methodology based on adaptive hierarchical refinement of NURBS, immersed boundary methods, and T-spline CAD surfaces. Comput Methods Appl Mech Eng.

[31] Burman E, Claus S, Hansbo P, Larson MG, Massing A (2015). CutFEM: discretizing geometry and partial differential equations. Int J Numer Meth Eng.

[32] Wassermann B, Kollmannsberger S, Bog T, Rank E (2017). From geometric design to numerical analysis: a direct approach using the finite cell method on constructive solid geometry. Comput Math Appl.

[33] Elfverson D, Larson MG, Larsson K (2018). CutIGA with basis function removal. Adv Model Simul Eng Sci.

[34] Casquero H, Bona-Casas C, Toshniwal D, Hughes TJ, Gomez H, Zhang YJ (2021). The divergence-conforming immersed boundary method: application to vesicle and capsule dynamics. J Comput Phys.

[35] Shephard MS, Georges MK (1991). Automatic three-dimensional mesh generation by the finite octree technique. Int J Numer Meth Eng.

[36] Abedian A, Parvizian J, Düster A, Khademyzadeh H, Rank E (2013). Performance of different integration schemes in facing discontinuities in the finite cell method. Int J Comput Methods.

[37] Kudela L, Zander N, Kollmannsberger S, Rank E (2016). Smart octrees: accurately integrating discontinuous functions in 3D. Comput Methods Appl Mech Eng.

[38] Petö M, Duvigneau F, Eisenträger S (2020). Enhanced numerical integration scheme based on image-compression techniques: application to fictitious domain methods. Adv Model Simul Eng Sci.

[39] Verhoosel C, van Zwieten G, van Rietbergen B, de Borst R (2015). Image-based goal-oriented adaptive isogeometric analysis with application to the micro-mechanical modeling of trabecular bone. Comput Methods Appl Mech Eng.

[40] Divi SC, Verhoosel CV, Auricchio F, Reali A, van Brummelen EH (2020). Error-estimate-based adaptive integration for immersed isogeometric analysis. Comput Math Appl.

[41] Joulaian M, Hubrich S, Düster A (2016). Numerical integration of discontinuities on arbitrary domains based on moment fitting. Comput Mech.

[42] Hubrich S, Stolfo PD, Kudela L, Kollmannsberger S, Rank E, Schröder A, Düster A (2017). Numerical integration of discontinuous functions: moment fitting and smart octree. Comput Mech.

[43] Hubrich S, Düster A (2019). Numerical integration for nonlinear problems of the finite cell method using an adaptive scheme based on moment fitting. Comput Math Appl.

[44] Bui H-G, Schillinger D, Meschke G (2020). Efficient cut-cell quadrature based on moment fitting for materially nonlinear analysis. Comput Methods Appl Mech Eng.

[45] Lasserre JB (1998). Integration on a convex polytope. Proc Am Math Soc.

[46] Gonzalez-Ochoa C, McCammon S, Peters J (1998). Computing moments of objects enclosed by piecewise polynomial surfaces. ACM Trans Graph.

[47] Mousavi SE, Sukumar N (2010). Numerical integration of polynomials and discontinuous functions on irregular convex polygons and polyhedrons. Comput Mech.

[48] Chin EB, Lasserre JB, Sukumar N (2015). Numerical integration of homogeneous functions on convex and nonconvex polygons and polyhedra. Comput Mech.

[49] Chin EB, Sukumar N (2020). An efficient method to integrate polynomials over polytopes and curved solids. Comput Aided Geom Des.

[50] Ventura G (2006). On the elimination of quadrature subcells for discontinuous functions in the extended finite-element method. Int J Numer Meth Eng.

[51] Duczek S, Gabbert U (2015). Efficient integration method for fictitious domain approaches. Comput Mech.

[52] Abedian A, Düster A (2019). Equivalent Legendre polynomials: numerical integration of discontinuous functions in the finite element methods. Comput Methods Appl Mech Eng.

[53] Müller B, Kummer F, Oberlack M (2013). Highly accurate surface and volume integration on implicit domains by means of moment-fitting. Int J Numer Meth Eng.

[54] Sudhakar Y, de Almeida JM, Wall WA (2014). An accurate, robust, and easy-to-implement method for integration over arbitrary polyhedra: application to embedded interface methods. J Comput Phys.

[55] Gunderman D, Weiss K, Evans JA (2021). High-accuracy mesh-free quadrature for trimmed parametric surfaces and volumes. Comput Aided Des.

[56] Gunderman D, Weiss K, Evans JA (2021). Spectral mesh-free quadrature for planar regions bounded by rational parametric curves. Comput Aided Des.

[57] Parvizian J, Düster A, Rank E (2007). Finite cell method. Comput Mech.

[58] Giannelli C, Jüttler B, Speleers H (2012). THB-splines: the truncated basis for hierarchical splines. Comput Aided Geometric Design.

[59] Bazilevs Y, Calo VM, Cottrell JA, Evans JA, Hughes TJR, Lipton S, Scott MA, Sederberg TW (2010). Isogeometric analysis using T-splines. Comput Methods Appl Mech Eng.

[60] Béchet E, Minnebo H, Moës N, Burgardt B (2005). Improved implementation and robustness study of the x-FEM for stress analysis around cracks. Int J Numer Meth Eng.

[61] de Prenter F, Verhoosel C, van Zwieten G, van Brummelen E (2017). Condition number analysis and preconditioning of the finite cell method. Comput Methods Appl Mech Eng.

[62] Buffa A, Puppi R, Vázquez R (2020). A minimal stabilization procedure for isogeometric methods on trimmed geometries. SIAM J Numer Anal.

[63] Hansbo A, Hansbo P (2002). An unfitted finite element method, based on Nitsche’s method, for elliptic interface problems. Comput Methods Appl Mech Eng.

[64] Ruess M, Schillinger D, Bazilevs Y, Varduhn V, Rank E (2013). Weakly enforced essential boundary conditions for NURBS-embedded and trimmed NURBS geometries on the basis of the finite cell method. Int J Numer Meth Eng.

[65] Pande S, Papadopoulos P, Babuška I (2021). A cut-cell finite element method for Poisson’s equation on arbitrary planar domains. Comput Methods Appl Mech Eng.

[66] Mantzaflaris A, Jüttler B (2015). Integration by interpolation and look-up for Galerkin-based isogeometric analysis. Comput Methods Appl Mech Eng.

[67] Antolin P, Buffa A, Martinelli M (2019). Isogeometric analysis on V-reps: first results. Comput Methods Appl Mech Eng.

[68] Borden MJ, Scott MA, Evans JA, Hughes TJR (2011). Isogeometric finite element data structures based on Bézier extraction of NURBS. Int J Numer Meth Eng.

[69] D’Angella D, Kollmannsberger S, Rank E, Reali A (2018). Multi-level Bézier extraction for hierarchical local refinement of isogeometric analysis. Comput Methods Appl Mech Eng.

[70] Scott MA, Borden MJ, Verhoosel CV, Sederberg TW, Hughes TJR (2011). Isogeometric finite element data structures based on Bézier extraction of T-splines. Int J Numer Meth Eng.

[71] Cohen E, Riesenfeld R. F, Elber G (2001) Geometric modeling with splines. Taylor & Francis Ltd

[72] Farin G (2001) Curves and surfaces for CAGD: a practical guide. Morgan Kaufmann Publ inc

[73] Piegl L, Tiller W (1997). The NURBS book.

[74] Requicha AA, Rossignac JR (1992). Solid modeling and beyond. IEEE Comput Graphics Appl.

[75] Braid I C (1973) Designing with volumes, Ph.D. thesis, University of Cambridge

[76] Antonietti PF, Houston P, Pennesi G (2018). Fast numerical integration on polytopic meshes with applications to discontinuous Galerkin finite element methods. J Sci Comput.

[77] SAS O. C (2018) Open CASCADE 7.3.0, http://www.opencascade.com (May)

[78] Elber G (2019) Irit 11 user’s manual. http://www.cs.technion.ac.il/~irit/

[79] Antolin P, Buffa A, Puppi R, Wei X (2021). Overlapping multipatch isogeometric method with minimal stabilization. SIAM J Sci Comput.

[80] Sukumar N, Moës N, Moran B, Belytschko T (2000). Extended finite element method for three-dimensional crack modelling. Int J Numer Meth Eng.

[81] Beirão da Veiga L, Brezzi F, Cangiani A, Manzini G, Marini LD, Russo A (2013). Basic principles of virtual element methods. Math Models Methods Appl Sci.

[82] Farouki R, Rajan V (1988). Algorithms for polynomials in Bernstein form. Comput Aided Geometric Design.

